# Receptor Tyrosine Kinase-Targeted Cancer Therapy

**DOI:** 10.3390/ijms19113491

**Published:** 2018-11-06

**Authors:** Toshimitsu Yamaoka, Sojiro Kusumoto, Koichi Ando, Motoi Ohba, Tohru Ohmori

**Affiliations:** 1 Advanced Cancer Translational Research Institute (Formerly, Institute of Molecular Oncology), Showa University, 1-5-8 Hatanodai, Shinagawa-ku, Tokyo 142-8555, Japan; moba@pharm.showa-u.ac.jp; 2 Division of Allergology and Respiratory Medicine, Department of Medicine, Showa University School of Medicine, 1-5-8 Hatanodai, Shinagawa-ku, Tokyo 142-8555, Japan; k-sojiro@med.showa-u.ac.jp (S.K.); koichi-a@med.showa-u.ac.jp (K.A.); ohmorit@med.showa-u.ac.jp (T.O.)

**Keywords:** receptor tyrosine kinase, molecular target inhibitors, resistance mechanisms

## Abstract

In the past two decades, several molecular targeted inhibitors have been developed and evaluated clinically to improve the survival of patients with cancer. Molecular targeted inhibitors inhibit the activities of pathogenic tyrosine kinases. Particularly, aberrant receptor tyrosine kinase (RTK) activation is a potential therapeutic target. An increased understanding of genetics, cellular biology and structural biology has led to the development of numerous important therapeutics. Pathogenic RTK mutations, deletions, translocations and amplification/over-expressions have been identified and are currently being examined for their roles in cancers. Therapies targeting RTKs are categorized as small-molecule inhibitors and monoclonal antibodies. Studies are underway to explore abnormalities in 20 types of RTK subfamilies in patients with cancer or other diseases. In this review, we describe representative RTKs important for developing cancer therapeutics and predicting or evaluated resistance mechanisms.

## 1. Introduction

Numerous drugs have been developed to target the receptor tyrosine kinase (RTK) family of growth factor receptors containing aberrations such as mutations, deletions, translocations and amplification/overexpression. The RTK family includes 58 types of receptors grouped into 20 subfamilies based on their kinase domain sequence; these receptors control fundamental cell behaviors such as cell proliferation, apoptosis and migration [1]. All RTKs contain an extracellular domain, containing the ligand-binding site, single transmembrane region and cytosolic domain, which includes the region with protein tyrosine kinase activity. These RTKs receive and transmit signals from the environment in nature. Binding of their ligands to each extracellular region leads to self-association which generally guides the cytosolic regions to form dimers, producing activated tyrosine kinases. Aberrant activation of RTKs can be caused by ligand-dependent or ligand-independent mechanisms. In colorectal cancer, epidermal growth factor receptor (EGFR) ligand aberrations, with amphiregulin, epiregulin and transforming growth factor (TGF)-α may act as prognostic indicators and predictive biomarkers of the response to anti-EGFR antibodies, such as cetuximab or panitumumab, even when wild-type KRAS is present. Oncogenic mutations, translocations and amplifications are also RTK aberrations. EGFR mutation in non-small cell lung cancer (NSCLC), RET mutation in medullary thyroid carcinoma, ALK translocation and ROS1 translocation in NSCLC and HER2 amplification in breast cancer have been reported and therapies have been established. Because of recent advances in genomics analyses, additional potential candidates are currently under evaluation in many clinical trials.

In this review, we discuss the recent development of molecular targeting drugs for RTK aberrations in cancers, including potential or established molecular targets of EGFR, HER2, ALK, ROS1, vascular endothelial growth factor receptor (VEGF(R)), MET, insulin-like growth factor 1 receptor (IGF1R) and fibroblast growth factor receptor (FGFR), focusing on therapies for individual patients with cancer.

## 2. Biology of RTKs and Link with Cancer

### 2.1. Classification and Characterization of RTKs

RTKs consist of an extracellular ligand-binding region, single transmembrane domain and cytoplasmic kinase domain [2]. Particularly, the extracellular domain in each class of receptors has a different structure and sequence, which defines its ligand specificity. Each receptor possesses different types and numbers of distinctive protein motifs, such as an immunoglobulin-like (Ig) domain, leucine-rich domain (L domain), cysteine-rich domain (CR domain), or fibronectin type 3 (Fn3)-like domain. The cytoplasmic domain includes the tyrosine kinase domain and the C-terminal region [1]. Some receptors have a kinase insert domain that divides the kinase domain when sequences of various lengths are inserted [3]. The C-terminal domains vary between RTK families, rendering specificity and diversity of downstream signals to cells. However, only insulin receptor family protein has α and β subunits and exists as a dimer independently of ligand binding.

### 2.2. Mechanisms of Activation

Although there are various types of RTKs, they have two characteristics in common; (1) dimerization after binding to their ligands and (2) auto-phosphorylation of tyrosine residues [4]. Binding of ligands to the extracellular domains of RTKs triggers enzyme activation. The monomeric and self-inactivated receptor undergoes a dynamic conformation change, resulting in the homo-/hetero-dimer formation and tyrosine kinase activity. RTKs auto-phosphorylate tyrosine residues in kinase domain and the C-terminal region, leading to the assembly of signaling molecules containing the Src homology 2 domain and phosphotyrosine-binding domain. These molecules include kinases (phosphatidylinositol 3-kinase (PI3K), SRC), adaptor proteins (SHC, GRB2), transcriptional factors (signal transducer and activator of transcription (STAT)), ubiquitin ligases and phospholipases (PLC-γ) They activate downstream signal cascades, such as the RAS/RAF/mitogen-activated kinase (MAP), PI3K/AKT/mammalian target of rapamycin (mTOR), PLC-γ/protein kinase C and Janus kinase (JAK)/STAT pathways. Ultimately, these signals induce diverse biological responses, that is, cell growth, survival, inhibition of apoptosis, promotion of angiogenesis and activation of cell motility [5].

### 2.3. EGFR/ERBB Family

Receptors for epidermal growth factor (EGF), leukocyte tyrosine kinase (LTK) (ALK), platelet-derived growth factor (PDGF), vascular endothelial growth factor (VEGF), fibroblast growth factor (FGF), hepatocyte growth factor (HGF) (MET) and insulin are significantly associated with oncogenic aberrations and are suitable targets for molecular-targeting therapy. Here, we provide detailed information about these six subfamilies of RTKs.

The EGFR/ERBB family is a representative target for cancer therapy to which small molecule tyrosine kinase inhibitors (TKIs) and monoclonal antibodies (mAbs) are developed, approved and applied. The EGFR/ERBB protein family is comprised of four structurally related kinases: EGFR, HER2, HER3 and HER4 [4]. These receptors form homo-/hetero dimers in response to ligand binding and each shows different specificity and affinity to its ligands; EGFR specifically binds to EGF, TGF-α, amphiregulin and epigen, both EGFR and HER4 bind to betacellulin, HB-EGF and epiregulin and HER3 and/or HER4 bind to the neuregulin family, EGF and TGF-α [6]. Based on data obtained using gene-deficient mice, loss of the *ErbB* family results in organ defects including the epidermis, mammary gland, lung, heart and brain, demonstrating their essential roles in organ development [7,8,9]. In contrast, transgenic mice models overexpressing *Erbb* showed the development and progression of solid tumors such as mammary adenocarcinomas, skin squamous cell carcinoma and lung adenocarcinoma [10].

#### 2.3.1. EGFR

In human cancer, several types of gene aberrations in the ERBB family have been reported. In glioma, an oncogenic mutation named as EGFRvIII (EGFR verIII) was first identified in 1988 [11]. This mutation is a deletion of EGFR exons 2–7, corresponding to the EGFR ectodomain, which enhances tumorigenicity via ligand-independent dimerization and constitutive activation. EGFRvIII has been detected not only in high-grade human gliomas but also in lung, breast, skin, head and neck and ovarian cancers. However, adequate therapeutic strategies with high efficacy have not been reported, despite the evaluation of specific antibodies, vaccines and EGFR TKIs in several clinical trials [12]. In lung adenocarcinoma, the most extensively investigated somatic mutations in EGFR are deletions in exon19 (dels746–750) or a replacement of leucine by arginine at codon 858 in exon 21 (L858R), leading to tremendous contributions to clinical practice [13,14]. These two types of mutations exist in the ATP-binding domain of the tyrosine kinase, yielding an increase of EGFR activity. EGFR overexpression is observed in 40–80% of NSCLC patients caused by epigenetic aberrations and gene copy number alterations. Also, several reports have shown the EGFR gene amplification in colorectal cancer (CRC) and squamous cell carcinoma of the head and neck (SCCHN), although EGFR mutations are less commonly detected [15,16]. Detailed information on EGFR-TKI targeting of these active mutations is described in Section 3.

#### 2.3.2. HER2

The primary HER2 aberration is gene amplification, resulting in protein overexpression. Approximately 1–37% of tumors show HER2 overexpression. Particularly, HER2 fulfills a crucial role in breast cancer as a driver mutation. Approximately 20% of patients with breast cancer harbor HER2 amplification; consequently, anti-HER2 drugs such as trastuzumab and lapatinib exhibit significant efficacy in patients with HER2-positive breast cancer [17]. Furthermore, HER2 gene amplification is detected in gastric or gastroesophageal cancers (6–30%), pancreas (2–29%) and bladder cancer (5–15%) [18]. HER2 has no binding ability to any known EGFR ligands owing to the lack of a ligand-binding domain. HER2 preferably form heterodimers with other EGFR family members via extracellular domains, leading to its constitutive activation. Of all four EGFR family receptors, heterodimer with HER3 exhibits the most robust kinase activity, thereby triggering the activation of downstream signals such as PI3K/AKT and MAPK pathway. Notably, HER2 homodimer formation is possible when HER2 is overexpressed.

#### 2.3.3. HER3

HER3 requires interactions with other EGFR members to exert its biological function because HER3 has weak or no intrinsic kinase activity. Other EGFR family proteins phosphorylate nine tyrosine phosphorylation sites in the intracellular domain of HER3. Among them, six tyrosine residues (Y1054, Y1197, Y1222, Y1260, Y1276 and Y1289) are binding sites for the PI3K/p85 regulatory subunit, resulting in the strong survival-promoting signal mediated by PI3K/AKT/mTOR pathway in cancer cells. In addition, phospho-Y1199 and Y1262 interact with GRB2 and phospho-Y1328 binds to SHC. These two adaptor proteins are involved in the growth signal via the MAPK pathway. In several types of cancer, HER3 functions as a tumorigenic molecule via interactions with HER2 and EGFR. Recently, however, somatic mutations also have been reported in several tumors including CRC (11%) and gastric cancer (12%). Many of these mutations are located in the extracellular domain (V104, A232, P262, G284 D297, G325 and T355), while two mutations were identified in the kinase domain (S846I and E928G).

### 2.4. Anaplastic Lymphoma Kinase (ALK)

ALK belongs to the LTK family containing a single glycine-rich domain in the extracellular region [19]. ALK is thought to regulate the development of the brain and nervous systems. ALK-deficient mice display aberrations in hippocampus formation [20]. In cancer development, however, ALK took the spotlight when it was discovered that the ALK gene is fused to echinoderm microtubule-associated protein like 4 (EML4) on chromosome 2 in NSCLC [21]. This was the first discovery of chromosomal rearrangement in solid tumors, which resulted in the clinical application of crizotinib to patients with NSCLC showing EML4/ALK fusion. Currently, several ALK-fusions have been identified in lung tumors, that is, ALK/KIF5B, ALK/TFG, ALK/ASXL2 [22,23]. Additionally, this gene was found to be mutated or amplified in neuroblastoma and glioblastoma [24,25,26]. Detailed information regarding ALK-targeted cancer therapy is described in another section.

### 2.5. VEGFR, PDGF/kit and FGF

VEGF, PDGF/kit and FGF contain seven, five and three Ig-like domains in the extracellular domain, respectively [27]. These RTKs have been implicated in vascular development by affecting the proliferation and migration of endothelial cells or pericytes. Among them, VEGF is a major regulator of tumor angiogenesis via endothelial cell proliferation and the permeability of blood vessels [28,29]. VEGF is expressed in most human cancers such as breast, kidney and colon and patients with tumors showing elevated VEGF expression have a poor prognosis [30]. The five genes comprising the VEGF family (VEGF-A, -B, -C, -D and placenta growth factor (PlGF)) exhibit 40–80% homology [31]. VEGF-A, generally referred to as VEGF, has some RNA splicing variants such as VEGF_121_, VEGF_165_, VEGF_189_ and VEGF_206_. Particularly, VEGF_121_ and VEGF_165_, which exist in the circulating blood, mainly regulate tumor neovascularization. These isoforms transfer the angiogenesis signal via two VEGF receptors, VEGFR-2 (KDR/Flk-1) and VEGFR-1 (flt-1) [30].

VEGFR2 is an essential molecule that initiates and positively controls the sprouting angiogenesis. VEGFR2 has five major auto-phosphorylation sites, that is, Y951 within the kinase insert domain, Y1054 and 1059 in the kinase catalytic domain and Y1175 and Y1214 in the carboxy-terminal domain. Phosphorylation of Y951 recruits the T cell-specific adapter, which mediates actin reorganization, cell migration and increased blood permeability. Phospho-Y1175 (pY1175) binds to PLC-γ and then activates growth signals through the PKCβ/MAPK pathway [32]. The adapter proteins SHB and SCK also interact with pY1175, resulting in increased activation of PI3K and focal adhesion kinase, resulting in survival signal generation and stress fiber and focal adhesion formation [33,34]. It is generally considered that VEGFR1 functions as a decoy receptor of VEGFR2 in angiogenesis during organ development. VEGFR1 has approximately 10-fold higher affinity to VEGF than VEGFR2 but exhibits a weaker signal compared to VEGFR2 [35]. The soluble form of VEGFR1, produced by the alternative splicing of VEGFR1, can trap VEGF in the blood fluid [36]. Furthermore, VEGFR1-deficient mice display embryonic lethality, accompanied by large, disorganized blood vessels and abnormal proliferation of hemangioblasts [37]. In contrast, several studies have suggested that VEGFR1 can promote angiogenesis and induce bone marrow-derived vascular endothelial progenitor cells in tumor tissues [38]. Therefore, its precise function remains unclear.

The PDGFR family, also named as class III RTKs, includes PDGFRα, PDGFRβ, KIT (CD117, stem cell factor receptor), colony-stimulating factor 1 receptor and Fms-like tyrosine kinase 3 [3,27]. Mutations in PDGFR family genes, particularly *PDGFRα* and *c-KIT*, are most frequently found in gastrointestinal stromal tumors (GISTs) [39,40,41]. Active mutations in *c-KIT* are detected in 80–90% of sporadic GISTs, mainly between L_550_ and R_588_ in exon 11 corresponding to the juxtamembrane domain. *PDGFRα* is mutated in approximately 10% of GISTs in exon 12 (V561D), 14 (N659L, N659Y) and 18 (D842V) which encode the cytoplasmic domain [42]. These gene mutations create a constitutively active form of the kinase that is not required for ligand binding to be activated. Approximately 20 families of inherited GISTs due to germline mutations in c-KIT or PDGFRα have been reported to date [43,44]. The mutation site is similar to cases of sporadic GIST, with the most common mutation in exon 11, 3 cases in exon 13 (L642E) and 3 cases in exon 17 (N822L, N822H) of c-KIT. Two mutations in *PDGFRα* were found in two families in exons 12 and 18.

*FGFR*s are comprised of four highly conserved genes (*FGFR1*–*4*) and one gene lacking the sequences corresponding to a kinase domain (*FGFR5*). Additionally, alternative splicing of these genes generates numerous variants of the receptors. Moreover, there are many FGFs (at least 22 members), several of which bind to one or more receptors. The characteristic feature of FGF/FGFR is its contribution to the diverse and complex functions and signaling of the FGF-axis which modulates tissue development, the endocrine system, homeostasis, angiogenesis and wound healing [45]. Numerous studies have detected genetic alterations of FGFR in human cancers, such as gene amplifications, activating mutations and chromosomal translocations. Gene amplification and protein overexpression of FGFR1 was detected in squamous cell lung carcinoma (~20%), small-cell lung carcinoma (6%) and breast cancers (10–13%), including hormone receptor-positive or triple-negative breast cancers [46,47,48]. FGFR2 is also amplified in gastric cancers (5–10%) [49] and a small subset of breast cancers [49]. Amplification of FGFR3 and 4 is not frequently reported. Somatic activating mutations in FGFRs, particularly FGFR2 and FGFR3, are commonly found in many types of tumors including NSCLCs and endometrial, gastric, ovarian and urothelial cancers [45]. Characteristically, mutations in FGFRs are mainly present in the extracellular and transmembrane domains, leading to increased affinity of FGFRs for their ligands (e.g., S252W in FGFR2, S373C and Y376C in FGFR2-IIIc, G370C and Y373C in FGFR3-IIIc) or constitutive receptor dimerization via aberrant disulfide bond formation [50]. Additionally, FGFR3 mutations in the extracellular domain (R248C, S249C) promote the dimerization of receptors and ligand-independent activation. Furthermore, somatic mutations in the kinase domain of FGFR1 (N546K, N549H/N) result in constitutively kinase activation of the receptors, although this is less frequent [51]. Third, oncogenic alterations in FGFRs result in fusion with other genes via chromosomal translocations. For example, in a small fraction of glioblastoma multiforme cases, *FGFR1* or *FGFR3* fuse in-frame with transforming acidic coiled-coil containing 1 (*TACC1*) or *TACC3*, respectively. TACC possesses a motif involved in protein oligomerization, causing the FGFR/TACC protein to display ligand-independent dimerization and constitutive activation. This fusion protein localizes to mitotic spindle poles, where it induces mitotic and chromosomal segregation defects and aneuploidy [52]. Additionally, FGFR2-fused proteins have been identified in intrahepatic cholangiocarcinomas, lung, thyroid and prostate cancers; that is, FGFR2/citron Rho-interacting kinase, FGFR2/coiled-coil domain-containing protein 6, FGFR2/cell cycle and apoptosis regulator protein 2 and FGFR2/oral-facial-digital syndrome 1 protein [45].

VEGFR, PDGF/kit and FGFR have similar structures in their intercellular kinase domains. Thus, multikinase inhibitors (MKIs) targeting these RTKs, for example, regorafenib, pazopanib and dovitinib, have been developed and approved. Such MKIs can concomitantly inhibit multiple signals evoked by different RTKs to increase efficacy and reduce resistance. However, they exhibit a variety of side effects, particularly dermatological reactions such as hand-foot skin syndrome, rash, pruritus and general gastrointestinal symptoms.

### 2.6. Hepatocyte Growth Factor (HGF)/Mesenchymal-Epithelial Transition Factor Receptor (MET)

c-MET was identified as a proto-oncogene in 1984 [53] and is a receptor for HGF. The HGF-MET axis regulates the embryogenesis, morphogenesis and development of the liver and placenta [54]. The immature MET protein is proteolytically cleaved to produce a 45-kDa α-subunit and 145-kDa β-subunit; these two subunits links through a disulfide bond to generate the mature receptor. The β-subunit contains one Sema domain and four Ig-domains in its extracellular region and tyrosine residues in its kinase domain (Y-1134, 1135) and C-terminal domain (Y-1349, 1356), which are essential for diverse cellular signaling events. Phospho-Y1356 interacts with GRB2, PLC-γ and PI3K. Y-1349 and Y1356 recruit SRC, SHC and GRB2-associated binding protein 1 (GAB1). GAB1 contains several tyrosine residues which become phosphorylated upon its interaction with MET. This results in the assembly of numerous signaling molecules, including PI3K, SHP2, CRK and PLC-γ. In human cancers, *MET* amplification and protein overexpression have been detected in gastric carcinoma [55], medulloblastomas [56] and NSCLCs with acquired resistance to EGFR-TKI [57]. Moreover, elevated expression of the MET protein via increased transcription results in the occurrence and progression of many types of tumors including thyroid [58], colorectal [59], pancreatic [60], ovarian [61] and breast cancers [62]. Activating point mutations in the kinase domain in MET have been reported in both sporadic and inherited renal papillary carcinomas [63].

### 2.7. Insulin/Insulin-Like Growth Factor (IGF) Receptor

Insulin receptor family members (class II) including insulin receptor (InsR) and IGF1R and are distinct from other RTK classes in their structures. The receptor is constituted by two α subunits and two β subunits and exists as a dimer even under in the absence of a ligand. Both the α and β subunits are derived from a single gene and mRNA. A functional receptor with α/β chains is created from the precursor protein through glycosylation, proteolytic cleavage and crosslinks between several cysteine residues. The α chains contain only the extracellular domain containing two L domains, one CR domain and one Fn3 domain, while the β subunit possesses the extra-, membrane- and intracellular kinase domains that transduce external signals inside the cells [64,65]. After auto-phosphorylation of three tyrosine residues (Y1146, Y1150 and Y1151 in InsR, Y1131, Y1135 and Y1151 in IGF1R) in the kinase domain of InsR family proteins, they recruit an adaptor protein, insulin receptor substrate 1 (IRS-1) [66]. InsR or IGF1R binds to IRS-1 via the phosphotyrosine-binding domain and phosphorylates multiple sites on tyrosine in IRS-1, leading to the association with several signaling molecules such as PI3K, GRB2 and SHP2 [67,68].

## 3. EGFR Targeted Cancer Therapy, Resistance, & Overcoming Resistance

### 3.1. Cancer Therapy Targeting EGFR

TKIs and mAbs are currently the main approaches for targeting EGFR in manifold human cancer therapies [69,70,71,72]. The mechanisms of these approaches involve targeting the intracellular tyrosine kinase domain (TKIs) and binding to extracellular domains [73,74]. Previous clinical studies revealed the significant efficacy of cancer therapies targeting EGFR in overall survival (OS), progression-free survival (PFS) and overall response (OR) in several types of cancers, including NSCLC, CRC, pancreatic cancer, breast cancer and SCCHN [75,76,77]. However, in most cases, EGFR TKIs lose their sensitivity within 9–14 months. Previous studies reported that several possible mechanisms of EGFR TKIs lead to acquired resistance [78].

#### 3.1.1. EGFR TKIs

**Figure 1:**
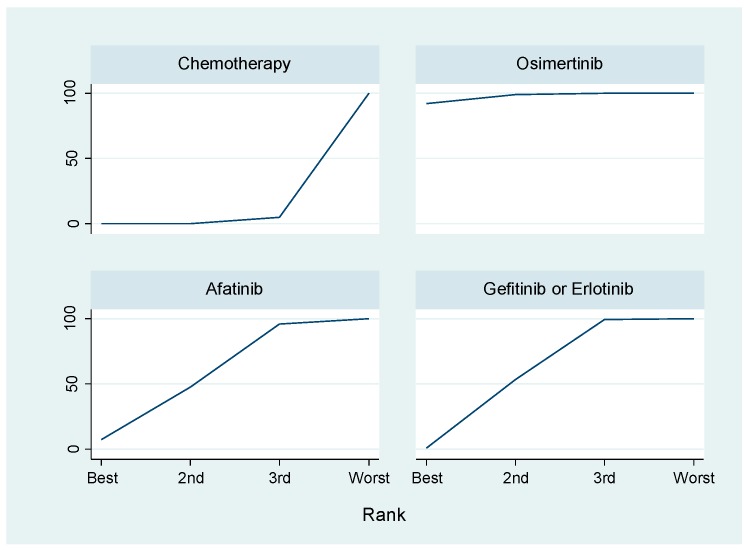
Network meta-analysis of PFS among chemotherapy, gefitinib or erlotinib, afatinib and osimertinib. Data are represented as the cumulative ranking curve. The SUCRA value means the ratio of the area of under the cumulative ranking curve to the total area in the plot and could be utilized to compare each treatment to an ideal treatment which is absolutely and systematically the best. Therefore, a larger SUCRA indicates more effective treatment in the present analysis. PFS; progression-free survival, SUCRA; surface under the cumulative ranking curve.

TKIs bind to ATP-binding pockets located at the intracellular catalytic kinase domain of RTKs, blocking activation of downstream signaling [79]. Although compared to traditional platinum-based combination chemotherapy, EGFR-TKIs provided significant clinical benefits and became a cornerstone treatment strategy for various cancers including NSCLC with *EGFR*-activating mutations, the low safety profiles of these agents should be acknowledged [80]. Skin rushes, such as acneiform eruptions, are often observed [81]. Notably, EGFR TKIs related pulmonary toxicities and interstitial lung disease have emerged as serious adverse effects. Although the mechanism remains unclear, previous studies revealed that male sex, a history of smoking and concomitant interstitial pneumonia and poor performance status were all significant risk factors for interstitial lung disease [82,83,84]. Several EGFR TKIs are currently available for cancer treatment [85]. Reversible, first-generation EGFR-TKIs (gefitinib and erlotinib) clinically improved the prognosis of patients with NSCLC harboring *EGFR*-activating mutations (exon 19 15-base pair deletion and exon 21 L858R) [86,87]. Irreversible, second-generation EGFR TKIs (afatinib and dacomitinib) showed an increased cellular potency against EGFR oncogenic variants (e.g., EGFR-L858R/T790M) [88,89,90,91,92]. Nevertheless, patients who respond to these treatments exhibit acquired resistance within 9–14 months. The secondary mutation T790M is detected in approximately 60% of these resistant cases [78,93,94,95]. To overcome T790M-mediated resistance, third-generation EGFR TKIs (osimertinib, olmutinib and rociletinib) have been developed. These agents target T790M, re-sensitizing cancer cells to EGFR TKI inhibition [93,96,97,98]. A recent report of AURA-3 in a randomized phase III clinical trial revealed that osimertinib represents the standard therapy in NSCLC with *EGFR*-activating mutation after the failure of first-line EGFR-TKIs compared to platinum-based chemotherapy [99]. Moreover, the FLAURA study demonstrated that osimertinib was more effective in improving PFS as a first-line treatment for *EGFR* mutation-positive advanced NSCLC compared to standard EGFR-TKIs (gefitinib or erlotinib) with a similar safety profile and lower rates of serious adverse events [100]. A recent network meta-analysis (NMA) revealed that osimertinib was the best therapeutic agent among five major EGFR TKIs as first-line treatments because it achieved the longest PFS of patients with NSCLC containing *EGFR*-activating mutations [101]. We performed a NMA using the statistical method of the frequentist approach [102] to evaluate four phase III trials [100,103,104,105] including 1305 patients for PFS to examine first-line treatment of NSCLC with *EGFR*-activating mutations. The statistical NMA method makes it possible to indirectly compare the efficacy or safety of treatments even if there were no previous reports of RCTs that compared the treatments. We compared osimertinib versus chemotherapy, gefitinib or erlotinib and afatinib using the statistical method of NMA. In this analysis, the primary endpoint was PFS. The data were expressed as mean differences (MD) and 95% confidence interval (CI). The result revealed that the PFS following treatment with osimertinib was significantly prolonged compared to that of chemotherapy (MD, 14.15; 95% CI, 5.46 to 22.83; *p* < 0.001) or gefitinib or erlotinib (MD, 7.94; 95% CI, 0.83 to 15.15; *p* = 0.029), although there was no significant difference in PFS between osimertinib and afatinib treatment (MD, 8.53; 95% CI, −2.62 to 19.33; *p* = 0.136) (Table 1). Next, we used the surface under the cumulative ranking curves (SUCRA), a popular ranking method, which considers the ratio of the area under the cumulative ranking curve to the total area in the plot, to identify which therapeutics had the best effects on PFS of patients with NSCLC harboring *EGFR*-activating mutations as a first-line therapy. The larger SUCRA indicates the more favorable treatment. The result revealed that osimertinib ranked higher than gefitinib or erlotinib and afatinib (Figure 1, Table 2). These results strongly indicate that osmertinib can be used as a therapeutic agent for NSCLC with *EGFR*-activating mutations as a first-line therapy. However, additional treatment strategies for NSCLC with *EGFR*-activating mutations should be developed and studies should be conducted to understand the resistance mechanisms to overcome resistance to first-, second- and third- EGFR TKIs.

#### 3.1.2. Anti-EGFR mAbs

Anti-EGFR mAbs were developed to specifically react against the EGFR extracellular region, leading to prevention of ligand binding and inhibition of receptor dimerization, autophosphorylation and downstream signaling. Further, these mAbs cause receptor dimerization, ubiquitination, degradation and prolonged downregulation [106,107]. EGFR family receptors are considered as therapeutic targets of mAbs to inhibit their activities in tumor growth and resistance. However, the efficacy for OS was limited because of resistance mechanisms from a clinical perspective. Several mAbs against EGFRs including cetuximab and panitumumab are currently available for CRC and SCCHN as cancer therapeutics. Cetuximab in combination with platinum-fluorouracil chemotherapy significantly improved OS compared to platinum-fluorouracil chemotherapy alone as the first-line treatment in patients with recurrent or metastatic SCCHN [108]. Combination therapy with panitumumab and FOLFOX4 resulted in significant improvements in OS compared to FOLFOX4 therapy alone in patients with metastatic CRC without *RAS* mutations [109]. Based on these results, cetuximab and panitumumab were clinically restricted to use in combination with standard chemotherapy but not as a single agent.

### 3.2. Resistance Mechanisms to EGFR TKIs and Overcoming Resistance

EGFR TKIs are clinically beneficial for patients with NSCLC harboring *EGFR*-activating mutations. However, the emergence of resistance to these anticancer drugs is nearly inevitable. To develop specific therapeutic agents for cancer treatment, a better understanding of the resistance mechanisms is required. Resistance mechanisms to EGFR TKIs can be divided into four categories: secondary mutation in *EGFR*, activation of alternative pathways, phenotypic transformation and resistance to apoptotic cell death. The most common cause of resistance to first-generation EGFR TKIs is T790M in *EGFR*, a secondary mutation [78,110].

#### 3.2.1. Secondary Mutation of *EGFR*

A secondary mutation in *EGFR*, T790M in exon 20, reduces the binding affinity of first-generation EGFR TKIs to EGFR by changing the protein conformation, resulting in resistance. Approximately 50–60% of patients with NSCLC treated with first-generation EGFR TKIs develop the T790M secondary mutation [110,111]. To overcome this resistance, second- (e.g., afatinib, dacomitinib and neratinib) and third- (osimertinib and olmutinib) generation EGFR TKIs have been developed. Although the clinical dose of afatinib cannot reach a concentration that will interfere with the T790M mutant EGFR, third-generation EGFR TKIs have specific inhibitory effects on activating mutations and the T790M mutation. The third-generation EGFR TKIs osimertinib and olmutinib showed a high objective response in 50–60% of patients with the T790M mutation [112,113,114]. Moreover, a recent phase III study demonstrated that osimertinib was more effective as a first-line treatment compared to first-generation EGFR-TKIs in terms of PFS [115]. A recent study reported a tertiary C797S mutation as the cause of resistance to third-generation EGFR TKIs [116]. To overcome C797S, the development of fourth-generation EGFR inhibitors is highly desirable. Although minor mutations, such as E709X, Ins19, Ins20, S681I and L861Q, have been detected with a low probability in clinical specimens and are considered to mediate resistance [117,118], the contributions of these mutations to EGFR TKI sensitivity are not completely understood. Patients with NSCLC with these minor mutations are rare and therefore studies analyzing large datasets based on the accumulation of clinical sensitivity and gene mutation are essential.

#### 3.2.2. Activation of Alternative Pathways

Other factors of EGFR resistance include conversion from EGFR to alternative signaling pathways. In this category, the most frequently observed resistance mechanism is related to the signaling HGF receptor, MET. Met signaling is hyper-activated by MET gene amplification or by an increased HGF supply via autocrine or paracrine signaling. EGFR signaling pathways maintain their activity even in EGFR TKI-resistant cancers caused by hyper-activated MET signaling [119]. Consequently, combination therapy with EGFR and MET TKIs are expected to overcome this resistance. Several clinical studies of combination therapies of MET TKIs (cabozantinib and INC280) and EGFR TKIs are currently underway [120,121]. A recent study reported the acquisition of dual resistance mechanisms in NSCLC harboring an *EGFR*-activating mutation to MET TKI and EGFR TKI following previous EGFR-TKI treatment [122]. These studies strongly suggest that a combination of EGFR TKI and MET TKI can overcome the acquired resistance to EGFR-TKI. A previous in vitro study demonstrated that this combined treatment was generally effective. Through MET-amplification by stepwise dose-escalation of gefitinib for 12 months, a cell line with acquired gefitinib-resistance was generated, named as PC-9MET1000 [123] and was investigated in detail. We previously reported acquired wild-type KRAS overexpression and attenuation of afatinib resistance following a drug holiday [124]. NSCLC cell lines with acquired resistance to gefitinib harboring reduced EGFR signaling increased the collateral sensitivity to tumor necrosis factor-α by autophosphorylation of EGFR with reduced AKT-phosphorylation [125]. These results strongly suggest that the understanding of heterogeneity of EGFR TKI resistance mechanisms will contribute to the development of more effective therapeutic strategies for patients with NSCLC. However, how acquired resistance mechanisms develop requires further examination by tissue biopsy or plasma analysis.

#### 3.2.3. Phenotypic Transformation

A previous study has reported that repeated bioptic sampling from patients with EGFR mutation revealed histological transformation from adenocarcinoma to small cell lung cancer [126]. Although the mechanisms underlying this phenomenon remains to be fully clarified, SCLC cells might arise from a minority of preexistent cells which were exposed to EGFR-TKIs and either derived from the multipotent stem cells, or from non-small cell lung cancer cells by transdifferentiating [111]. Another common mechanism of phenotypic transformation is epithelial-mesenchymal transition (EMT), a process characterized by the loss of polarity and cell to cell contact by the epithelial cell layers, which undergo a remarkable cytoskeleton remodeling [127]. A previous study reported that during the process of EMT, AXL upregulation might be a significant mechanism of acquired resistance to EGFR-TKI in EGFR-active mutant NSCLCs [128].

#### 3.2.4. Resistance to Apoptotic Cell Death

For EGFR-TKIs, down-regulation of cellular apoptosis may be a possible cause of cancer cell resistance. NF-κB is a transcription regulator that tune tumor cell growth and proliferation, leading to the resistance to apoptotic cell death. Previous experimental studies had revealed that activation of NF-κB signaling pathway could confer TKI resistance in EGFR mutant NSCLC cells [129]. Furthermore, the investigators also reported that inhibition of NF-κB signaling could cause TKI sensitivity in EGFR-mutant NSCLC cells and that upregulated NF-κB signaling condition was relative to worse PFS and decreased OS in EGFR-active mutant NSCLC in patients treated with EGFR-TKIs [130].

### 3.3. Resistance Mechanisms to Anti-EGFR mAbs and Overcoming Resistance

Previous studies have revealed various resistance mechanisms. KRAS and NRAS mutations in exon 2 were reported to lead to resistance to anti-EGFR mAbs in CRC. Alternative pathways reported to contribute to resistance to anti-EGFR mAbs include bypass signaling activation which causes gene amplification or mutation in RTKs (HER2, FGFR1, MET and PDGFR) and activation of downstream signaling pathways caused by mutations in NRAS, BRAF, or PI3CA, or deletion in PTEN [131,132]. Previous studies strongly indicated that combination chemotherapies of anti-EGFR antibodies with EGFR-TKIs, anti-HGF antibodies and MEK inhibitors can overcome this resistance [133,134,135].

## 4. HER2 Targeted Cancer Therapy, Resistance, & Overcoming Resistance

### 4.1. Cancer Therapies Targeting HER2

To date, five Her2-targeted therapeutics are clinically available. Among them, three therapeutics are humanized mAbs that specifically bind to the Her2 extracellular domain: trastuzumab, pertuzumab and trastuzumab emtansine. Trastuzumab and pertuzumab have different binding epitopes. Trastuzumab binds to Her2 extracellular domain IV to mediate antibody dependent cell-mediated cytotoxicity (ADCC) [136], suppress downstream signaling [137] and downregulate this receptor [138,139]. Pertuzumab binds to domain II and prevents heterodimer formation with the other Her family member [140]. Trastuzumab emtansine is an antibody-drug conjugate that uses trastuzumab as a delivery carrier of the cytotoxic drug emtansin for Her2-positive cancer. Lapatinib and neratinib are small molecules TKIs approved for Her2-positive breast cancer. These drugs respectively reversibly and irreversibly inhibit both EGFR and Her2 by preventing ATP-binding to the receptors [141,142]. Although these Her2-targeted therapeutics inhibit tumor growth and improve the outcomes of cancer patients, the emergence of resistance to these drugs is inevitable.

### 4.2. Resistance & Overcoming Resistance to Anti-HER2 Therapies

#### 4.2.1. Obstacles in Drug Binding to Her2

The resistance mechanisms of the Her2-targeted therapeutics include the following: (1) obstacles to drug binding to Her2, (2) emergence of bypass signaling and (3) failure of host ADCC response.

Most factors preventing drug binding to Her2 are mediated by alterations in the Her2 protein. The 185-kDa Her2 protein gradually loses its extracellular domain by proteolytic shedding and the remaining membrane-associated 95-kDa fragment (p95Her2) acquires constitutive activity [143]. Higher p95Her2 expression was reported in patients with node-positive breast cancer [144]. This form of Her2 lacks a binding site for trastuzumab and is associated with clinical resistance to this therapeutic [145]. In contrast, high expression of p95Her2 in cancer shows good responses to lapatinib [145]. Several mutations in the Her2 kinase domain contribute to the sensitivity to small-molecule Her2 inhibitors. Mutations in this domain were observed in 2–5% of various cancers [146,147,148]. Among these mutations, the Her2 T798I gatekeeper mutation is associated with a high level of resistance to lapatinib [149] and neratinib [150] because of the lower binding affinity for these drugs. The emergence of the T798I mutation was confirmed upon clinical progression of neratinib-treated patients with breast cancer. Interestingly, the Her2 L869R mutation is a gain-of-function mutation and increases the sensitivity to niratinib [150]. An EGFR/Her2/VEGFR TKI, tesevatinib [149] and afatinib [150] are expected to overcome this Her2 T798I-mediated resistance. It has also been reported that the loss of Her2 amplification during neoadjuvant trastuzumab treatment is related to poor outcomes of patients compared to patients with Her2-retained tumors [151].

#### 4.2.2. Emergence of Bypass Signaling

As with other TKIs, the emergence of bypass signaling that compensates for the Her2 signaling pathway induces resistance to Her2-TKIs. Bypass signaling is mediated by the emergence of alternative RTKs and/or by downstream molecule activation regardless of Her2 regulation. Activation of other members of ErbB/Her family receptors can amplify Her2 signaling by heterodimer formation and compensate for this signaling. It has been reported that inhibition of Her2 caused compensatory activation of downstream molecules mediated by Her3 up-regulation and resulted in induction of resistance to lapatinib [152]. Moreover, because trastuzumab cannot prevent Her2/Her3 dimerization, up-regulation of Her3 also induces resistance to this drug [153]. In RTKs other than ErbB/Her family members, amplifications of Met [154,155], Axl [156] and IGF-1R [157] are related to resistance. These receptors can also induce resistance to EGFR-TKIs [158,159,160], suggesting they are major alternative bypass signaling escape methods from ErbB/Her family receptors in cancer. The downstream molecules related to Her2-TKIs resistance include PI3K/Akt/mTOR and Src family non-receptor tyrosine kinase. PI3K is a phospholipid kinase that transmits signals from RTK to Akt by producing phosphatidylinositol (3,4,5)-trisphosphate (PIP3). A mutant p85a PI3K regulatory subunit is frequently observed in many cancers and causes constitutive activation of this enzyme [161,162]. A PIP3 phosphatase, PTEN, normally inhibits PI3K-mediated signaling. However, the loss of PTEN function is frequently observed in cancer which occurs through PTEN gene mutations or transcriptional regulation [163]. These alterations cause Akt pathway activation unrelated to upstream RTK and reduce the cancer sensitivity to Her2-TKIs [137,164]. Src family kinases (SFKs) such as c-Src, Yes, Fyn, Fgr, Yrk, Lyn, Blk, Hck and Lck interact with many transmembrane proteins including RTK and transmit signals to downstream molecules by tyrosine residue phosphorylation [165]. The activation of SFKs by gene mutation or overexpression has been frequently observed in many types of cancer and is related to the survival, angiogenesis, proliferation and invasion of the tumor [166]. Up-regulation of SFKs have been reported to mediate lapatinib resistance [167]. Numerous kinase inhibitors have been developed and researchers are entering an era in which drugs to overcome respective Her2-TKIs resistances are being developed. As in other forms of RKI-resistance, it is important that these inhibitors can be used in combination with Her2-TKIs for the remaining Her2 signaling after acquiring resistance.

#### 4.2.3. Failure of Host ADCC Response

Among the classified groups, ADCC response failure was reported only in cases with trastuzumab resistance. Fc gamma receptors (FCGRs) enable immune effector cells to bind to antigen-bound IgG antibodies in an ADCC reaction. A nonsynonymous single-nucleotide polymorphism (SNP) in FCGRIIa (amino acid 131 changed from histidine (H) to arginine (R)) mediates lower binding affinity to IgG2 [168], while an SNP in FCGRIIIa (amino acid 158 changed from phenylalanine (F) to valine (V)) mediates efficient binding affinity to IgG1 [169]. As a result, these SNPs modulate the ADCC response to trastuzumab. In patients with Her2-positive metastatic breast cancer, the clinical response to trastuzumab in patients with peripheral blood mononuclear cells harboring FCGRIIa H or FCGRIIIa V alleles was significantly better than when these cells harbored the other types of alleles [170,171,172]. These results suggest that systemic immune reaction systems in hostal cancer patients contribute to the efficacy of trastuzumab.

## 5. ALK Targeted Cancer Therapy, Resistance, & Overcoming Resistance

To target the ALK fusion protein which is expressed in approximately 5% of NSCLCs, first- to third-generation ALK-TKIs have been developed. The superiority of alectinib against crizotinib was reported as a first-line therapy for ALK-positive NSCLC. It was suggested that the 2nd and additional biopsies (including liquid biopsy) and analysis of ALK mutations would become more important when selecting optimal ALK-TKIs for cancers continuously acquiring mutations.

### 5.1. Targeting of ALK Fusion Protein

ALK participates in brain and nerve development and is expressed in the nervous system, testicles and small intestine in adults [173]. Like other receptor tyrosine kinases, wild-type ALK is activated by the binding of its ligand (such as augmentor α), whereas, mutant ALK is located in the cytosol and is constitutively activated in many cancer cells, such as anaplastic large cell lymphoma, in a ligand-independent manner [174,175]. In 2007, Soda et al. discovered the EML4-ALK fusion gene, which acts as an oncogene and contributes to cancer progression in NSCLC [21]. *EML4-ALK* is expressed in 3–5% of NSCLC cases [176] and crizotinib, an inhibitor of ALK, c-MET and ROS1, was granted accelerated approval by the Food and Drug Administration (FDA) in 2011 as a therapeutic drug for ALK-positive NSCLC. Crizotinib significantly prolonged PFS and was superior to cytotoxic chemotherapy in chemonaïve advanced ALK-rearranged NSCLC (PFS: crizotinib 10.9 m versus chemotherapy 7.0 m, HR: 0.45 (95% CI 0.35–0.6), *p* < 0.0001) and has been considered as standard chemotherapy [177]. However, even if cancer patients show a good initial response to crizotinib treatment, most cases become resistant to this drug within 1–2 years of administration.

### 5.2. ALK TKI Resistance & Overcoming Resistance

**Figure 2:**
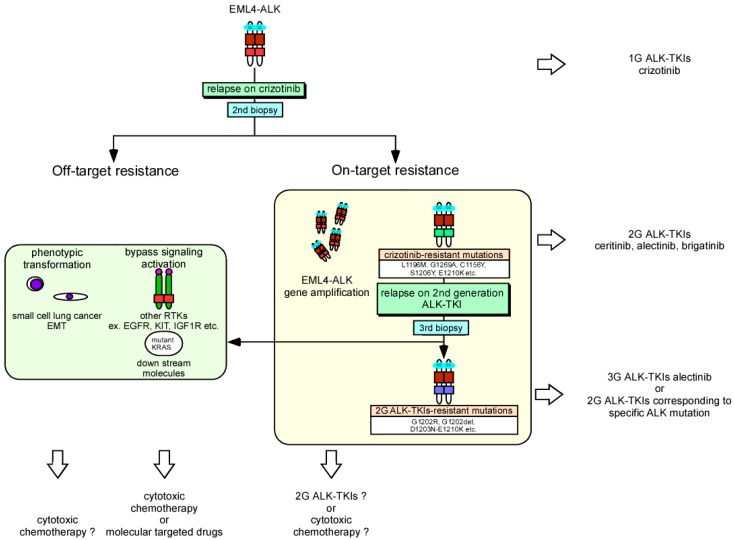
Forecast map of the treatment sequence for ALK-positive NSCLC.

The molecular mechanisms of crizotinib-resistance are largely divided into 2 groups: on-target resistance (ALK gene alteration) and off-target resistance (Figure 2). In the off-target group, bypass signaling activation (such as the other RTKs: EGFR, KIT, IGF1R and downstream signaling molecules; SRC, MEK/ERK) [178,179,180] and morphological alterations (small cell lung cancer and epithelial-mesenchymal transition) contribute to this resistance [181,182,183]. Recently, a specific inhibitor (SHP099) to Src homology 2-containing protein tyrosine phosphatase 2 (SHP-2, PTPN11), which contributes to a wide range of bypass signaling activations, was developed. Combination therapy of this drug and ALK-TKIs is expected to restore the ALK-TKI resistance [184]. Alterations in the ALK gene are found in 30–40% of ALK-TKI-resistant patients with NSCLC; among these alterations, ALK gene amplification is observed in 6–18% of cases [178,185]. In contrast to the T790M mutation which is the primary EGFR mutation causing first-generation EGFR-TKI resistance, more than 10 ALK mutations including the gatekeeper mutation L1196M, C1156, G1269 and so forth, are related to crizotinib resistance and occur at equal frequencies [186]. Ceritinib and alectinib were developed as second-generation ALK-TKIs for cases of crizotinib-resistance and were approved as breakthrough therapies by the FDA in 2013 and 2014, respectively. These drugs had antitumor effects against NSCLC harboring many resistant ALK mutations and showed a more than 50% of response rate even in crizotinib-treated patients. Moreover, crizotinib is a substrate of the drug transporter p-glycoprotein and thought to be eliminated by the blood-brain barrier [187]. Weak delivery of this drug to the central nervous system greatly limits the treatment of metastatic brain tumors, while both second-generation ALK-TKIs were shown to be effective for treating central nervous system tumors in clinical studies compared to crizotinib [188,189]. Recently, two clinical phase III studies (global study: ALEX [189,190] and in Japan: J-ALEX [191]) were performed to compare alectinib and crizotinib as the first-line treatment of patients with ALK-positive NSCLC who had not received prior systemic therapy for metastatic disease. Although the dosage of alectinib differed in these studies, both studies demonstrated an improvement in PFS compared to crizotinib (ALEX: estimated median PFS: 25.7 m versus 10.4 m, HR: 0.53 (95% CI: 0.38–0.73; *p* < 0.0001), J-ALEX: estimated median PFS: not reached versus 10.2 m, HR: 0.34 (95% CI: 0.17–0.71; *p* < 0.0001)) and occurrence of serious adverse events (grade ≥ 3) was lower in the alectinib group than in the crizotinib group (ALEX: 41% vs. 50%, J-ALEX: 26% vs. 52%). These results suggest that a prolonged response duration can be achieved by taking countermeasures to the resistance mechanism during the primary treatment of cancer. Based on these observations, ceritinib and alectinib were approved by the FDA as the first-line therapeutics for ALK-positive metastatic NSCLC in 2017. However, because these clinical studies have not completed the analysis of OS time, it remains controversial whether a primary choice of second-generation ALK-TKIs or sequential usage of these drugs following crizotinib achieves the best survival benefit. Additionally, resistance mechanisms to second-generation ALK-TKIs were analyzed using clinical materials. Gainor et al. reported that more than 50% of cases of resistance were caused by additional ALK gene mutations [182]. Thus, resistant mutations in ALK are more commonly observed for second-generation ALK-TKIs than for crizotinib. The most common ALK mutations were G1202R and F1174C/L and among them, G1202R caused resistance to all second-generation ALK-TKIs including ceritinib and alectinib. Interestingly, several ALK mutations were found to mediate drug-specific resistance among second-generation ALK-TKIs and a third-generation ALK-TKI, lorlatinib, showed efficacy to all series of resistance mutations. Clinical trials of new-generation ALK-TKIs including brigatinib and lorlatinib are being promoted [192,193] which will increase the number of available therapeutics against ALK-positive NSCLC. Repeat biopsies including liquid biopsy and ALK-gene analysis during disease progression will become more important for choosing optimal ALK-TKIs. According to what mentioned above, a forecast map of the treatment sequence for ALK-positive NSCLC is presented, when crizotinib was used as the first-line treatment (Figure 2).

## 6. VEGF(R) Targeted Cancer Therapy, Resistance, & Overcoming Resistance

### 6.1. Targeted Therapy to Tyrosine Kinase Domains Including VEGFRs

Targeted agents to the tyrosine kinase domains of VEGFRs have also been developed. Approved oral small-molecule TKIs targeting VEGFR function as multi-kinase inhibitors (MKIs) have many targets besides VEGFR, such as FGFRs, PDGFRs and other RTKs associated with angiogenesis. Many agents, such as sorafenib, sunitinib, regorafenib, pazopanib, axitinib, cabozantinib and vandetanib, have been approved for advanced or recurrent solid tumors, including renal cell carcinoma (RCC), hepatocellular carcinoma (HCC), medullary thyroid carcinoma, GIST and have antitumor effects when administered as monotherapy [194]. Approved drugs for tyrosine kinase domains including VEGFRs are listed in Table 3.

### 6.2. Targeted Therapy to VEGF Family and Their Receptors

The first developed humanized mAb to VEGF was bevacizumab. This agent targets VEGF-A to prevent its binding to VEGFR-1 and -2. Bevacizumab is widely used to treat brain glioblastoma as a monotherapy or is combined with chemotherapy. Although promising preclinical data have been obtained, monotherapy with this agent did not show the expected effect except on brain glioblastoma. Chemotherapy combined with bevacizumab was demonstrated to exert antitumor effects as well as prolong survival among patients with advanced cancer including metastatic CRC (mCRC), NSCLC and ovarian cancer [195,196,197]. Ramucirumab is a humanized mAb to VEGFR2 and has antitumor activity when combined with chemotherapy. Phase III trials of advanced gastric cancer, mCRC and NSCLC showed that addition of ramucirumab to standard chemotherapy prolonged survival compared to chemotherapy alone [198,199,200]. Aflibercept is a fully humanized recombinant fusion protein composed of the extracellular domains of VEGFR-1 and -2 fused to the Fc portion of human immunoglobulin G1. It binds to VEGF-A, VEGF-B, PlGF-1 and PlGF-2, functions as a decoy VEGFR and prevents these ligands from binding to and activating their receptors. In clinical trials of mCRC, standard chemotherapy combined with aflibercept significantly improved survival compared to chemotherapy alone [201]. The approved drugs for targeting VEGFRs are listed in Table 4.

### 6.3. Resistance Mechanisms to Anti-VEGF(R) Therapies

Despite the initial response, resistance to anti-VEGF or VEGFR therapy is ultimately inevitable. The mechanisms of resistance to anti-VEGF therapies have been investigated. Although the VEGF-VEGFR axis is the primary factor in angiogenesis, various other components are involved. Blockade of the VEGF-VEGFR axis may alter its effects on other pathways to maintain angiogenesis. Preclinical investigations have indicated that VEGFR2 blockade induced upregulation of other proangiogenic factors including FGF family members, angiopoietin (ANG), PDGF and HGF [194,202].

#### 6.3.1. FGF(R)

Anti-VEGF therapy induced upregulation of FGF and dual blockage of VEGF and FGF had an inhibitory effect on angiogenesis and tumor progression in a preclinical model [202], the efficacy is limited in the clinic. The small-molecule inhibitor dovitinib, which targets both FGFR and VEGF, was assessed in patients with progressive advanced renal cell carcinoma (RCC) after anti-VEGF therapies. The best OR and PFS were only 3.6% and 3.7 months, respectively [203]. A phase III trial comparing dovitinib with sorafenib in patients with advanced RCC who had progressed on previous VEGF-targeted therapies and mTOR therapies failed to show a survival benefit of dovitinib [204]. Further biomarker analysis is required for select patients who may respond to these TKIs and more potent agents and/or combination therapies should be developed for these patients in the future.

#### 6.3.2. ANG and TIE2

The ANG/TIE2 receptor axis is a well-known cascade of angiogenesis. An investigational report suggested that tumor progression during anti-VEGF therapies is associated with upregulation of ANG/TIE2 and dual blockade of VEGF/VEGFR and ANG/TIE2 could restore drug sensitivity in tumor cells resistant to anti-VEGF therapy [205]. Trebananib is a small-molecule inhibitor of angiopoietin-1/2 and clinical trials to assess the effect of this agent combined with anti-VEGF agents (bevacizumab, pazopanib, sorafenib, sunitinib) for various solid malignancies are ongoing [206].

#### 6.3.3. PDGF(R)

The interaction between PDGF(R) and VEGFR has been well-described. Based on this interaction, an alternative pathway acting through PDGF-PDGFR can quickly be developed for anti-VEGF therapy and this alteration confers resistance to anti-VEGF therapy [207]. Simultaneous blocking of PDGFR and VEGFR may be promising for treating cases resistant to anti-VEGF therapy. However, clinically, it is unclear whether upregulation of the PDGF-PDGFR axis confers resistance to anti-VEGF(R) therapy [194].

#### 6.3.4. HGF and MET

A mouse model of glioblastoma revealed that inhibition of VEGF signaling with bevacizumab promoted MET activation by recruiting phosphatase PTP1B to a VEGFR2/MET heterocomplex, after which malignant cells acquired resistance to anti-VEGF therapy. In this model, combined MET and VEGF inhibition decreased tumor invasion and increased survival. Moreover, bevacizumab-resistant human glioblastoma tissue exhibited MET activation along with epithelial-mesenchymal transition-like features. These findings suggest that the upregulation of MET leads to resistance to anti-VEGF therapy. Therefore, combined therapy targeting both VEGF-VEGFR and HGF/MET should overcome anti-VEGF therapy resistance [208]. Cabozantinib is an orally administered small-molecule inhibitor targeting VEGFR, MET and AXL. Among patients with advanced renal cell carcinoma who are resistant to VEGFR TKI treatment, cabozantinib showed superior efficacy to standard therapy with the mTOR inhibitor everolimus [209]. However, the objective response and PFS were 17% and 7.4 months, respectively, which are not satisfactory.

## 7. Other RTK-Targeted Cancer Therapies: MET/FGF(R)/IGF1R

### 7.1. MET

Aberrant cMET activation occurs through HGF-independent mechanisms such as MET mutation, gene amplification and transcriptional upregulation [210]. There are two types of therapeutics for inhibiting cMET tyrosine kinase activity: neutralizing antibodies targeting the cMET-HGF axis and small-molecule inhibitors for preventing tyrosine phosphorylation. Several TKIs and mAbs targeting cMET have been evaluated in clinical studies. Unfortunately, no MET TKIs or mAbs have been approved for clinical application. However, MET amplification, overexpression, mutation (particularly exon 14 skipping mutation) are potential candidates and MET inhibitors for the clearly validated predictive biomarkers are urgently needed. The published phase III studies of MET inhibitors are listed below (Table 5).

#### 7.1.1. MET TKIs

MET TKIs can be divided into three types (I, II and III) with different binding modes and selectivity profiles. The pro-MET kinase adapts a unique autoinhibitory conformation with the activation loop locked into the ATP triphosphate binding site via a salt bridge between D1228 and K1110. Type I MET-inhibitors are ATP-competitive and bind this MET unique autoinhibitory conformation by interacting with Y1230 in the MET activation loop. Type I inhibitors can be further divided into types Ia and Ib. Type Ia inhibitors interact with Y1230, the hinge and the solvent front glycine residue G1163 (analog to the same position as ALK G1202 and ROS1 G2032), whereas type Ib inhibitors have stronger interactions with Y1230 and the hinge but no interaction with G1163. Therefore, type Ib inhibitors are specific for MET compared to type Ia inhibitors. Type II inhibitors are multitarget MET inhibitors and ATP-competitive, which pass the gatekeeper and occupy the ATP-binding pocket. Type III inhibitors bind to allosteric sites distinct from the ATP-binding site, which are not tested in clinical trials for oncology.

Crizotinib, approved for treating ALK-positive NSCLC, is a type Ia inhibitor, with potent inhibitory activity towards cMET and ALK and ROS1 [214]. Cabozantinib, which has been approved for treating medullary thyroid cancer, is a type II inhibitor with multi-targeted tyrosine kinase for cMET, VEGFR2, AXL, KIT, TIE2, Fms-like tyrosine kinase 3 and RET [218]. These two inhibitors are currently approved and used clinically. Other MET TKIs are under evaluation in clinical trials, such as type Ib: capmatinib, tepotinib, savolitinib and AMG337 and type II: glesatinib, merestinib.

#### 7.1.2. MET mAbs

Onartuzumab is a humanized one-armed mAb against cMET and in phase III clinical trials for unselected NSCLC patients with erlotinib plus onartuzumab vs. erlotinib plus placebo. Erlotinib plus onartuzumab did not improve clinical outcomes [212]. Taken together, onartuzumab plus standard therapy in several phase II and III trials of gastroesophageal adenocarcinoma, breast cancer, glioblastoma and colorectal carcinoma has exhibited disappointing results. These findings suggest that ligand-blocking antibodies are not effective for MET inhibition. Rilotumumab is a humanized mAb for neutralizing HGF to prevent the formation of the HGF-cMET axis [211] and has been evaluated in clinical trials.

#### 7.1.3. Resistance to MET Inhibitors

Several resistance mechanisms to MET TKIs have been reported including (a) MET amplification [219], point mutations in MET [220] and MET over-expression [221], (b) KRAS amplification [219], (c) bypass signaling activation [222] and (d) altered miRNA expression [223].

### 7.2. FGF(R)

Although no anti-cancer agents have been approved for treating cancer patients with FGFR aberrations, numerous clinical trials are currently underway. The therapies for inhibiting FGFR activation include small-molecule TKIs targeting several growth factor receptors (include FGFR), multi-target TKIs (non-selective FGFR TKIs) and selectively targeted FGFR kinase domain, selective FGFR TKIs and mAbs against FGFR and FGFR ligand traps. The published phase III studies of FGF(R) inhibitors are listed below. (Table 6).

#### 7.2.1. Non-Selective FGFR TKIs

Phylogenetically, the kinase domain of FGFR, VEGFR and PDGFR families are related and TKIs have been developed as an inhibitor of VEGFRs and as inhibitors of both FGFRs and PDGFR, as multitarget TKIs. Dovitinib (TKI258) is a non-selective TKI targeting VEGFR1-3, FGFR1-3 and PDGFR and has been evaluated in phase III clinical trials. An open-label, multicenter phase III study compared dovitinib to sorafenib as a third-line targeted therapy for metastatic RCC. Dovitinib showed clinical activity for treating advanced RCC following VEGF-targeted and mTOR inhibitor therapy but was not superior to sorafenib [204]. Nintedanib (BIBF1120) received FDA approval for treating patients with idiopathic pulmonary fibrosis, a rare disease but not for in applications in oncology. Lucitanib (E3810) and ponatinib (AP24534) are under clinical evaluation.

#### 7.2.2. Selective FGFR TKIs

To increase on-target effectiveness and reduce toxic effects induced by multitarget TKIs, selective inhibitors for FGFRs have been developed. FGFR1–3 have highly similar structures and thus selective FGFR TKIs inhibit FGFR1–3. Even in early clinical trials of selective FGFR TKIs, successful treatment of patients with FGFR fusions and FGFR2 amplification was observed, while unclear results were observed for other FGFR aberrations. For example, in cancers with *FGFR1* amplification, only one patient responded to AZD4547, an FGFR1–3 inhibitor, among 20 patients with FGFR1-amplified squamous NSCLC [225]. Another FGFR1–3 inhibitor, NVP-BGJ398, caused tumor regression in only one patient with FGFR1-amplified breast cancer in a phase I study [226]. Additionally, cancers with *FGFR3* mutation respond infrequently to this treatment. In contrast, patients with FGFR2 amplification appear to respond well to selective FGFR inhibitors. Three of nine FGFR2-amplified patients with gastric cancer responded to AZD4547 [225]. FGFR fusions, such as FGFR2-BICC1 gene fusion in cholangiocarcinoma and HCC and FGFR3-TACC3 fusion in advanced solid tumors, showed good responses to NVP-BGJ398 and JNJ-42756493, respectively, even in early phase clinical studies [226,227]. LY2874455, TAS120 and Debio-1347 are currently in phase I trials.

#### 7.2.3. FGFR mAbs and FGF Ligand Traps

Limited clinical evidence currently is available and several monoclonal antibodies against FGFRs are only in early clinical trials. MGFR1877S is a humanized anti-FGFR3 mAb. FP-1039 is a soluble fusion protein consisting of the FGFR1-IIIc splice isoform and functions as a ligand trap. FPA144 is an FGFR2-IIIb blocking mAb. These mAbs are being evaluated in early phase clinical trials [228].

#### 7.2.4. Resistance to FGFR Inhibitors

In vitro studies revealed gatekeeper mutations in FGFRs and the activation of alternative RTKs, enabling bypass of downstream signaling activation. Gatekeeper mutations in FGFRs occur in the ATP binding cleft. FGFR3_V555M, FGFR1_V561 and FGFR2_V564 induce resistance to FGFR inhibitors in vitro [229,230]. These gatekeeper mutations may create a steric conflict to limit drug-binding efficacy. Therefore, to overcoming these resistance mutations, irreversible covalent FGFRs have been developed [231]. The ERBB receptor family and other RTK activators have been reported as bypass-resistance mechanisms [232].

### 7.3. IGF1R

IGF1R is required for malignant transformation and low IGF bioactivity protects against the development of neoplasms [64,233]. Therefore, increased IGF1R signaling is linked with an elevated cancer risk and induces more aggressive behaviors of cancers. There are three strategies for inhibiting IGF1R signaling with clinical applications: IGF1R-targeted monoclonal antibodies, IGF ligand-neutralizing monoclonal antibodies and small-molecule TKIs. Some of these agents are under clinical evaluation. Numerous drug candidates have been synthesized to target IGF1R and have shown significant activity in preclinical studies. However, in phase III clinical trials, the outcomes were not promising and, unfortunately, there are no FDA-approved IGF1R inhibitors. The critical fault of IGF1R inhibitor is the lack of a predictive biomarker such as ERBB2-positive breast cancer for trastuzumab or ALK-fusion protein-driven NSCLC for crizotinib. Candidate predictive markers have been reported such as circulating IGF-1 levels in the serum, IGF1R expression in the tissues and nuclear IGF1R expression [234,235,236]; therefore, it is necessary for clinical trials to identify and validate appropriate clinical applications of IGF1R blockade. The published phase III studies of IGF1R inhibitors are listed below. (Table 7).

#### 7.3.1. IGF1R-Targeted mAbs and IGF Ligand-Neutralizing mAbs

IGF1R-targeted monoclonal antibodies, such as figitumumab, cixutumumab, ganitumab, dalotuzumab, robatumumab, AMG-479 and BIIB022 have been evaluated in clinical trials for solid tumors. IGF ligand-neutralizing monoclonal antibodies such as Medi-573 and BI836845 are also under clinical evaluation. Unfortunately, two phase III studies revealed that figitumumab failed to improve OS in patients with advanced non-adenocarcinoma NSCLC treated with erlotinib and carboplatin/paclitaxel, respectively [237,238].

#### 7.3.2. IGF1R TKIs

Small-molecule inhibitors of IGF1R are in clinical trials, such as BMS-754807, linsitinib, XL228, AXL1717, PL225B and KW-2450. Linsitinib has been tested in combination with erlotinib in patients with NSCLC with EGFR-activating mutations and resulted in inferior outcomes compared to erlotinib alone in a phase II study [241]. Furthermore, linsitinib did not improve OS compared to placebo in patients with advanced adrenocortical carcinoma [240]. Further studies of IGF1R TKIs are required to identify predictive biomarkers, which may lead to improved therapeutics.

#### 7.3.3. Resistance to IGF1R inhibitors

Several factors may result in resistance to IGF1R inhibitors. InsR activation [242] and a strong autocrine loop of IGF1R/InsR may mediate IGF1R inhibitor resistance. An increased level of insulin, IGF1, or growth hormone may limit IGF1R inhibition.

### 7.4. c-KIT

Gain-of-function mutations in *c-KIT* gene altered the hyperactivation of RTK and are associated with several human malignancies, such as gastrointestinal stromal tumors (GISTs), acute myeloid leukemia (AML), mast cell leukemia (MCL) and melanoma. Imatinib dramatically improves the responses to the treatment of chronic myeloid leukemia (CML) and GISTs. Imatinib inhibits the tyrosine kinase activity of BCR-ABL and also mutated c-KIT and PDGFR. The clinical testing of imatinib in patients with melanoma, AML or MCL has been reported; however, the responses have been limited to a subset of patients carrying mutations in the *c-KIT* gene [243,244,245]. Therefore, the current clinical application for c-KIT inhibitor is restrained to GIST.

#### 7.4.1. c-KIT-TKI, Imatinib

GIST is the most common sarcoma accounting for 18% of all sarcomas. The 70–80% of GISTs harbor mutations in the *c-KIT* gene, commonly occurring within the juxtamembrane domain of exon 11 (70%) and in the extracellular domain of exon 9 (12%). Less frequently, *c-Kit* mutations are located in the kinase I domain of exon 13 (1%), or in the activation loop of exon 17 (1%) [246]. While standard chemotherapies are usually not helpful, imatinib is highly effective in delaying the progression and prognosis in patients with metastatic, unresectable, or recurrent GISTs [247]. The B2222 trial, the first large trial to show the effect of 1st-line imatinib in patients with advanced GISTs, revealed high objective response rates of 49% and durable disease control of 81%. Furthermore, the estimated 9-years OS rate for all patients was 35%. The median PFS is 17 and 25 months in c-kit exon 9 and exon 11 mutations, respectively [248]. Therefore, within 2–3 years of imatinib treatment, the majority of patients develop resistance [249]. The second-line therapy with sunitinib, which is MTI targeting VEGFR-1, VEGFR-2, fetal liver tyrosine kinase receptor 3 (FLT3), KIT, PDGFRα and PDGFRβ, has been found to prolong time to progression compared to placebo control from 6.4 to 27.3 weeks [250]. Third-line therapy with regorafenib also prolonged progression-free survival from 0.9 to 4.8 months [251].

#### 7.4.2. Resistance Mechanisms to c-KIT-TKI, Imatinib and Overcoming Resistance

Approximately 10–15% of patients with GIST do not respond to imatinib. Due to intrinsic or primary resistance, the treatment fails within the first 6 month. The tumors, showing intrinsic resistance to imatinib, carry the wild-type *c-KIT* and *PDGFRA*, exon 9 mutation in *c-KIT* and D842V substitution in *PDGFRA*. The function of wild-type c-KIT and PDGFRA might be replaced by alternate signaling pathways. The exon 9 mutation in c-KIT changes the conformation to less binding to imatinib. The D842V mutation in PDGFRA strongly alters the active conformation of PDGFRA, which is then unable to bind imatinib.

##### Secondary Mutations in c-KIT

The secondary *c-KIT* mutations determined either in the ATP binding pocket of the kinase domain (exon 13 and 14), or the kinase activation loop (exon 17 and 18). The T670I “gatekeeper” mutation in exon14 of ATP binding pocket directly inhibits imatinib binding [252]. Because imatinib is able to bind and inhibit only the non-activated conformation of KIT, the activation loop mutations induce imatinib resistance. Sunitinib remains sensitive to the most common secondary mutation of V654A in exon 13 and T670I in exon 14 but has no significant activity against the activation loop mutation in exon 17 and 18. Sunitinib also can only inhibit the inactive form of KIT.

##### Genomic Amplification of c-KIT

Amplification of c-KIT is not a common resistance mechanism [249]. The loss of wild-type allele in *c-KIT* mutation forms hemizygous, which has been correlated with more aggressive disease [253].

##### Loss of c-KIT Expression and the Alternative Signaling Activation

Interestingly, secondary *c-KIT* mutations have not been identified in GIST carrying wild-type KIT or PDGFRA. A *BRAF* V600E was determined in imatinib-resistance tissue, which did not express KIT and PDGFRA [254]. The oncogenic receptor tyrosine kinase, AXL, was overexpressed with c-KIT downregulation in imatinib-resistance cell line [255] and also AXL overexpression was detected in the tissue of imatinib-resistant patients by immunohistochemistry. The activation of focal adhesion kinase (FAK) appeared to be maintained in imatinib-resistant c-KIT mutant cells. Hence, FAK inhibitor could be a potential alternative strategy for imatinib-resistant GISTs [256]. *IGF1R* is amplified and overexpressed in GISTs that lack *c-KIT* or *PDGFR*α mutations and its inhibition may overcome imatinib-resistance [257]. The activation of alternative RTK with loss of c-KIT or PDGFRA mutation, named RTK switch, was induced by imatinib treatment. Other RTK switches induced by imatinib were identified in c-MET and FGFR. Furthermore, regarding the treatment with imatinib, FGFR-mediated reactivation of MAPK attenuates antitumor effect in patient-derived xenograft and the combination of KIT and FGFR inhibition might lead to increase efficacy [258]. Indeed, several clinical trials evaluating single or combination therapies are ongoing.

## 8. RTKs on Cancer Therapy

Over 80 kinds of molecular target drugs have been developed and approved worldwide. Out of these, 47 drugs target tyrosine kinase activity. Monoclonal antibodies account for 8 drugs, including anti-HER2 antibodies of trastuzumab, trastuzumab emtansine and panitumumab, anti-EGFR antibodies of cetuximab, panitumumab and necitumumab, anti-VEGFR2 antibody of ramucirumab and anti-PDGFRα antibody of olaratumab. Other 39 drugs are categorized as small-molecule inhibitor. Ten drugs are multikinase inhibitors (MTIs), including sorafenib, sunitinib, pazopanib, vandetanib, axitinib, regorafenib, cabozantinib, nintedanib, lenvatinib and midostaurin. Eighteen drugs are tyrosine kinase inhibitors, targeting proteins produced by oncogenes such as Bcr-Abl of imatinib, dasatinib, nilotinib, bosutinib, ponatinib, EGFR and/or HER2 of gefitinib, erlotinib, osimertinib, lapatinib, afatinib and neratinib, ALK of crizitinib, ceritinib, alectinib and brigatinib, JAK of ruxolitinib, Btk of ibrutinib and acalabrutinib. Nine drugs are serine/threonine kinase inhibitor, including temsirolimus and everolimus for targeting mTOR and vemurafenib and dabrafenib for targeting BRAF (V600E), trametinib and cobimetinib for targeting MEK and palbociclib, ribociclib and abemaciclib for targeting CDK4/6. Finally, 2 drugs, namely idelalisib and copanlisib, target PI3-kinase. The success of developing molecular targeting drugs has far exceeded that of classical chemotherapeutic agents, such as platinum, anti-metabolites, anti-tubulin agents. Among the molecular target drugs, the drugs targeting RTKs are the largest class and provide the most promising therapeutic effects for cancer patients.

## 9. Conclusions

In recent decades, through advances in genomic technologies, our understanding of RTKs and RTK-targeted therapies have dramatically progressed. Several drugs have been developed and approved for treating cancers by activating RTKs, including small-molecule inhibitors and mAbs. Although approved TKIs cause tumor regression and/or prolong survival, side-effects can occur because of the lack of selectivity to an individual target and the acquisition of drug resistance. Furthermore, for currently approved monoclonal antibodies, the efficacy of a single agent is very limited and therefore these monoclonal antibodies are used in combination with conventional chemotherapeutic agents. Targeted inhibitors to RTK fail to cure patients with cancer and thus these inhibitors are incomplete. Therefore, it is important to further examine the acquired resistance mechanisms to develop novel therapeutic strategies for tumor recurrence.

## Figures and Tables

**Table 1:** Network meta-analysis of PFS: osimertinib vs. chemotherapy, vs. gefitinib or erlotinib, vs. afatinib.

		MDs	SE	95% CIs	*p*-Value
	Chemotherapy	14.15	4.43	5.46–22.83	<0.001
Osimertinib vs.	Gefitinib/Erlotinib	7.94	3.62	0.83–15.15	0.029
	Afatinib	8.53	5.6	−2.62–19.33	0.136

Data are represented as MD of PFS (month) and 95% CIs. MDs; mean differences, PFS; progression-free survival, 95% CIs; 95% confidence intervals.

**Table 2:** Network meta-analysis in PFS among chemotherapy, gefitinib or erlotinib, afatinib and osimertinib.

Treatment	SUCRA	PrBest	MeanRank
Chemotherapy	2.7	0.1	3.9
Gefitininb/Erlotinib	48.4	0.6	2.5
Afatinib	50.6	4.4	2.5
Osimertinib	98.3	95.0	1.1

Data are represented as the SUCRA value, PrBest and MeanRank. The SUCRA value means the ratio of the area of under the cumulative ranking curve to the total area in the plot and could be utilized to compare each treatment to an ideal treatment which is absolutely and systematically the best. Therefore, a larger SUCRA indicates more effective treatment in the present analysis. PFS; progression-free survival, SUCRA; surface under the cumulative ranking curve, PrBest; the probability of the best treatment.

**Table 3:** Targeted therapy to tyrosine kinase domain including VEGFR.

Drugs	Sorafenib	Sunitinib	Regorafenib	Pazopanib	Axitinib	Cabozantinib	Vandetanib
Targets	VEGFR, PDGFR, c-KIT, FLT-3, RET	VEGFR, PDGFR, c-KIT, FLT-3, RET	VEGFR, PDGFR, FGFR, TIE2, c-KIT, FLT-3, RET	VEGFR, PDGFR, FGFR, c-KIT	VEGFR, PDGFR, c-KIT	VEGFR, c-MET, RET, TIE2, FLT-3, RET, AXL	VEGFR, EGFR, RET, TIE2, SRC
Clinical Indications	HCC ^a^, RCC ^b^, Thyroid carcinoma	GIST ^c^, RCC	mCRC ^d^, GIST	RCC, STS ^e^	RCC	MTC ^f^	MTC

^a^ HCC: hepatocellular carcinoma, ^b^ RCC: renal cell cancer, ^c^ GIST: gastrointestinal stromal tumor, ^d^ mCRC: metastatic colorectal cancer, ^e^ STS: soft-tissue sarcoma, ^f^ MTC: medullary thyroid cancer.

**Table 4:** Targeted therapy of VEGF family and their receptors.

Drug	Bevacizumab	Ramucirumab	Aflibercept
Target	VEGF-A	VEGFR2	VEGFR
Clinical Indications	Glioblastoma, mCRC ^1^, NSCLC ^2^, Ovarian cancer	Gastric cancer, mCRC ^1^, NSCLC ^2^	mCRC ^1^

^1^ mCRC: metastatic colorectal cancer, ^2^ NSCLC: non-small cell lung cancer.

**Table 5:** Published phase III studies of MET inhibitors.

Agents	Phase	Disease Characteristics	Comparison	Clinical Trial ID
*HGF antibodies*				
Rilotumumab	III	MET-positive G/GEJ cancer ^a^	Chemo ± Rilotumumab	NCT01697072 [211]
*MET antibodies*				
onartuzumab	III	MET-positive NSCLC ^b^	Erlotinib ± onartumumab	NCT01456325 [212]
onartuzumab	III	HER2(-)/MET(+)-GEC ^c^	mFOLFOX6 ± onartumumab	NCT01662869 [213]
*MET TKI*				
Crizotinib	III	ALK (+)-NSCLC	Chemo vs. crizotinib	NCT00932893 [214]
Crizotinib	III	ALK (+)-NSCLC	Chemo vs. crizotinib	NCT01154140 [177]
Crizotinib	III	ALK (+)-NSCLC	Alectinib vs. crizotinib	NCT02075840 [189]
Cabozantinib	III	HCC ^d^	Cabozantinib vs. Placebo	NCT01908426 [215]
Cabozantinib	III	RCC ^e^	Cabozantinib vs. Everolimus	NCT01865747 [216]
Cabozantinib	III	mCRPC ^f^	Cabozantinib vs. Prednisone	NCT01605227 [217]

^a^ G/GEJ: gastric or gastro-esophageal junction, ^b^ NSCLC: non-small cell lung cancer, ^c^ GEC: gastroesophageal adenocarcinoma, ^d^ HCC: hepatocellular carcinoma, ^e^ RCC: renal cell carcinoma, mCRPC: ^f^ metastatic castration-resistant prostate cancer.

**Table 6:** Published phase III studies of FGF(R) inhibitors.

Agents	Phase	Disease Characteristics	Comparison	Clinical Trial ID
*Non-selective*				
Dovitinib	III	RCC ^a^	Dovitinib vs. Sorafenib	NCT01223027 [204]
Ponatinib	III	CML ^b^	Ponatinib vs. Imatinib	NCT01650805 [224]

^a^ RCC: renal cell carcinoma, ^b^ CML: chronic myelogenous leukemia.

**Table 7:** Published phase III studies of IGF1R inhibitors.

Agents	Phase	Disease Characteristics	Comparison	Clinical Trial ID
*IGF-1R mAbs*				
figitumumab	III	NSCLC ^a^	chemo ± figitumumab	NCT00596830 [237]
figitumumab	III	NSCLC	erlotinib ± figitumumab	NCT00673049 [238]
ganitumab	III	Pancreatic adenocarcinoma	gemcitabine ± ganitumab	NCT01231347 [239]
*IGF-1R TKI*				
linsitinib	III	Adrenocortical carcinoma	linsitinib vs. Placebo	NCT00924989 [240]

^a^ NSCLC: non-small cell lung cancer.

## References

[1] Robinson D.R., Wu Y.M., Lin S.F. (2000). The protein tyrosine kinase family of the human genome. Oncogene.

[2] Schlessinger J. (2000). Cell signaling by receptor tyrosine kinases. Cell.

[3] Ullrich A., Schlessinger J. (1990). Signal transduction by receptors with tyrosine kinase activity. Cell.

[4] Yarden Y., Sliwkowski M.X. (2001). Untangling the erbb signalling network. Nat. Rev. Mol. Cell Biol..

[5] Yarden Y., Pines G. (2012). The ERBB network: At last, cancer therapy meets systems biology. Nat. Rev. Cancer.

[6] Arteaga C.L., Engelman J.A. (2014). Erbb receptors: From oncogene discovery to basic science to mechanism-based cancer therapeutics. Cancer Cell.

[7] Threadgill D.W., Dlugosz A.A., Hansen L.A., Tennenbaum T., Lichti U., Yee D., LaMantia C., Mourton T., Herrup K., Harris R.C. (1995). Targeted disruption of mouse egf receptor: Effect of genetic background on mutant phenotype. Science.

[8] Miettinen P.J., Berger J.E., Meneses J., Phung Y., Pedersen R.A., Werb Z., Derynck R. (1995). Epithelial immaturity and multiorgan failure in mice lacking epidermal growth factor receptor. Nature.

[9] Sibilia M., Wagner E.F. (1995). Strain-dependent epithelial defects in mice lacking the EGF receptor. Science.

[10] Muller W.J., Arteaga C.L., Muthuswamy S.K., Siegel P.M., Webster M.A., Cardiff R.D., Meise K.S., Li F., Halter S.A., Coffey R.J. (1996). Synergistic interaction of the NEU proto-oncogene product and transforming growth factor alpha in the mammary epithelium of transgenic mice. Mol. Cell. Biol..

[11] Yamazaki H., Fukui Y., Ueyama Y., Tamaoki N., Kawamoto T., Taniguchi S., Shibuya M. (1988). Amplification of the structurally and functionally altered epidermal growth factor receptor gene (c-ERBB) in human brain tumors. Mol. Cell. Biol..

[12] Reardon D.A., Wen P.Y., Mellinghoff I.K. (2014). Targeted molecular therapies against epidermal growth factor receptor: Past experiences and challenges. Neurol. Oncol..

[13] Lynch T.J., Bell D.W., Sordella R., Gurubhagavatula S., Okimoto R.A., Brannigan B.W., Harris P.L., Haserlat S.M., Supko J.G., Haluska F.G. (2004). Activating mutations in the epidermal growth factor receptor underlying responsiveness of non-small-cell lung cancer to GEFITINIB. N. Engl. J. Med..

[14] Paez J.G., Janne P.A., Lee J.C., Tracy S., Greulich H., Gabriel S., Herman P., Kaye F.J., Lindeman N., Boggon T.J. (2004). Egfr mutations in lung cancer: Correlation with clinical response to GEFITINIB therapy. Science.

[15] Barber T.D., Vogelstein B., Kinzler K.W., Velculescu V.E. (2004). Somatic mutations of EGFR in colorectal cancers and GLIOBLASTOMAS. N. Engl. J. Med..

[16] Jiang Z., Li C., Li F., Wang X. (2013). Egfr gene copy number as a prognostic marker in colorectal cancer patients treated with cetuximab or panitumumab: A systematic review and meta analysis. PLoS ONE.

[17] Verma S., Miles D., Gianni L., Krop I.E., Welslau M., Baselga J., Pegram M., Oh D.Y., Dieras V., Guardino E. (2012). Trastuzumab emtansine for her2-positive advanced breast cancer. N. Engl. J. Med..

[18] Apicella M., Corso S., Giordano S. (2017). Targeted therapies for gastric cancer: Failures and hopes from clinical trials. Oncotarget.

[19] Morris S.W., Naeve C., Mathew P., James P.L., Kirstein M.N., Cui X., Witte D.P. (1997). Alk, the chromosome 2 gene locus altered by the t(2;5) in non-hodgkin’s lymphoma, encodes a novel neural receptor tyrosine kinase that is highly related to leukocyte tyrosine kinase (LTK). Oncogene.

[20] Nakamura E., Kadomatsu K., Yuasa S., Muramatsu H., Mamiya T., Nabeshima T., Fan Q.W., Ishiguro K., Igakura T., Matsubara S. (1998). Disruption of the midkine gene (MDK) resulted in altered expression of a calcium binding protein in the hippocampus of infant mice and their abnormal behaviour. Genes Cells.

[21] Soda M., Choi Y.L., Enomoto M., Takada S., Yamashita Y., Ishikawa S., Fujiwara S., Watanabe H., Kurashina K., Hatanaka H. (2007). Identification of the transforming eml4-alk fusion gene in non-small-cell lung cancer. Nature.

[22] Rikova K., Guo A., Zeng Q., Possemato A., Yu J., Haack H., Nardone J., Lee K., Reeves C., Li Y. (2007). Global survey of phosphotyrosine signaling identifies oncogenic kinases in lung cancer. Cell.

[23] Rosenbaum J.N., Bloom R., Forys J.T., Hiken J., Armstrong J.R., Branson J., McNulty S., Velu P.D., Pepin K., Abel H. (2018). Genomic heterogeneity of alk fusion breakpoints in non-small-cell lung cancer. Mod. Pathol..

[24] Chen Y., Takita J., Choi Y.L., Kato M., Ohira M., Sanada M., Wang L., Soda M., Kikuchi A., Igarashi T. (2008). Oncogenic mutations of ALK kinase in neuroblastoma. Nature.

[25] Mosse Y.P., Laudenslager M., Longo L., Cole K.A., Wood A., Attiyeh E.F., Laquaglia M.J., Sennett R., Lynch J.E., Perri P. (2008). Identification of ALK as a major familial neuroblastoma predisposition gene. Nature.

[26] Dirks W.G., Fahnrich S., Lis Y., Becker E., MacLeod R.A., Drexler H.G. (2002). Expression and functional analysis of the anaplastic lymphoma kinase (ALK) gene in tumor cell lines. Int. J. Cancer.

[27] Blume-Jensen P., Hunter T. (2001). Oncogenic kinase signalling. Nature.

[28] DiSalvo J., Bayne M.L., Conn G., Kwok P.W., Trivedi P.G., Soderman D.D., Palisi T.M., Sullivan K.A., Thomas K.A. (1995). Purification and characterization of a naturally occurring vascular endothelial growth factor.Placenta growth factor heterodimer. J. Biol. Chem..

[29] Senger D.R., Galli S.J., Dvorak A.M., Perruzzi C.A., Harvey V.S., Dvorak H.F. (1983). Tumor cells secrete a vascular permeability factor that promotes accumulation of ascites fluid. Science.

[30] Ferrara N. (2002). Vegf and the quest for tumour angiogenesis factors. Nat. Rev. Cancer.

[31] Kerbel R.S. (2008). Tumor angiogenesis. N. Engl. J. Med..

[32] Matsumoto T., Bohman S., Dixelius J., Berge T., Dimberg A., Magnusson P., Wang L., Wikner C., Qi J.H., Wernstedt C. (2005). Vegf receptor-2 y951 signaling and a role for the adapter molecule tsad in tumor angiogenesis. EMBO J..

[33] Warner A.J., Lopez-Dee J., Knight E.L., Feramisco J.R., Prigent S.A. (2000). The shc-related adaptor protein, sck, forms a complex with the vascular-endothelial-growth-factor receptor KDR in transfected cells. Biochem. J..

[34] Holmqvist K., Cross M.J., Rolny C., Hagerkvist R., Rahimi N., Matsumoto T., Claesson-Welsh L., Welsh M. (2004). The adaptor protein SHB binds to tyrosine 1175 in vascular endothelial growth factor (VEGF) receptor-2 and regulates VEGF-dependent cellular migration. J. Biol. Chem..

[35] Ito N., Wernstedt C., Engstrom U., Claesson-Welsh L. (1998). Identification of vascular endothelial growth factor receptor-1 tyrosine phosphorylation sites and binding of sh2 domain-containing molecules. J. Biol. Chem..

[36] Kendall R.L., Wang G., Thomas K.A. (1996). Identification of a natural soluble form of the vascular endothelial growth factor receptor, flt-1, and its heterodimerization with KDR. Biochem. Biophys. Res. Commun..

[37] Fong G.H., Rossant J., Gertsenstein M., Breitman M.L. (1995). Role of the flt-1 receptor tyrosine kinase in regulating the assembly of vascular endothelium. Nature.

[38] Schwartz J.D., Rowinsky E.K., Youssoufian H., Pytowski B., Wu Y. (2010). Vascular endothelial growth factor receptor-1 in human cancer: Concise review and rationale for development of imc-18f1 (human antibody targeting vascular endothelial growth factor receptor-1). Cancer.

[39] Hirota S., Isozaki K., Moriyama Y., Hashimoto K., Nishida T., Ishiguro S., Kawano K., Hanada M., Kurata A., Takeda M. (1998). Gain-of-function mutations of c-kit in human gastrointestinal stromal tumors. Science.

[40] Hirota S., Ohashi A., Nishida T., Isozaki K., Kinoshita K., Shinomura Y., Kitamura Y. (2003). Gain-of-function mutations of platelet-derived growth factor receptor alpha gene in gastrointestinal stromal tumors. Gastroenterology.

[41] Corless C.L., Barnett C.M., Heinrich M.C. (2011). Gastrointestinal stromal tumours: Origin and molecular oncology. Nat. Rev. Cancer.

[42] Heinrich M.C., Corless C.L., Duensing A., McGreevey L., Chen C.J., Joseph N., Singer S., Griffith D.J., Haley A., Town A. (2003). Pdgfra activating mutations in gastrointestinal stromal tumors. Science.

[43] Nishida T., Hirota S., Taniguchi M., Hashimoto K., Isozaki K., Nakamura H., Kanakura Y., Tanaka T., Takabayashi A., Matsuda H. (1998). Familial gastrointestinal stromal tumours with germline mutation of the kit gene. Nat. Genet..

[44] Hirota S., Nishida T., Isozaki K., Taniguchi M., Nishikawa K., Ohashi A., Takabayashi A., Obayashi T., Okuno T., Kinoshita K. (2002). Familial gastrointestinal stromal tumors associated with dysphagia and novel type germline mutation of kit gene. Gastroenterology.

[45] Babina I.S., Turner N.C. (2017). Advances and challenges in targeting fgfr signalling in cancer. Nat. Rev. Cancer.

[46] Dutt A., Ramos A.H., Hammerman P.S., Mermel C., Cho J., Sharifnia T., Chande A., Tanaka K.E., Stransky N., Greulich H. (2011). Inhibitor-sensitive fgfr1 amplification in human non-small cell lung cancer. PLoS ONE.

[47] Peifer M., Fernandez-Cuesta L., Sos M.L., George J., Seidel D., Kasper L.H., Plenker D., Leenders F., Sun R., Zander T. (2012). Integrative genome analyses identify key somatic driver mutations of small-cell lung cancer. Nat. Genet..

[48] Reis-Filho J.S., Simpson P.T., Turner N.C., Lambros M.B., Jones C., Mackay A., Grigoriadis A., Sarrio D., Savage K., Dexter T. (2006). Fgfr1 emerges as a potential therapeutic target for lobular breast carcinomas. Clin. Cancer Res..

[49] Matsumoto K., Arao T., Hamaguchi T., Shimada Y., Kato K., Oda I., Taniguchi H., Koizumi F., Yanagihara K., Sasaki H. (2012). Fgfr2 gene amplification and clinicopathological features in gastric cancer. Br. J. Cancer.

[50] Wilkie A.O., Patey S.J., Kan S.H., van den Ouweland A.M., Hamel B.C. (2002). Fgfs, their receptors, and human limb malformations: Clinical and molecular correlations. Am. J. Med. Genet..

[51] Hart K.C., Robertson S.C., Kanemitsu M.Y., Meyer A.N., Tynan J.A., Donoghue D.J. (2000). Transformation and stat activation by derivatives of fgfr1, fgfr3, and fgfr4. Oncogene.

[52] Singh D., Chan J.M., Zoppoli P., Niola F., Sullivan R., Castano A., Liu E.M., Reichel J., Porrati P., Pellegatta S. (2012). Transforming fusions of fgfr and tacc genes in human glioblastoma. Science.

[53] Cooper C.S., Park M., Blair D.G., Tainsky M.A., Huebner K., Croce C.M., Vande Woude G.F. (1984). Molecular cloning of a new transforming gene from a chemically transformed human cell line. Nature.

[54] Schmidt C., Bladt F., Goedecke S., Brinkmann V., Zschiesche W., Sharpe M., Gherardi E., Birchmeier C. (1995). Scatter factor/hepatocyte growth factor is essential for liver development. Nature.

[55] Hara T., Ooi A., Kobayashi M., Mai M., Yanagihara K., Nakanishi I. (1998). Amplification of C-MYC, K-SAM, and c-met in gastric cancers: Detection by fluorescence in situ hybridization. Lab. Investig. J. Tech. Methods Pathol..

[56] Tong C.Y., Hui A.B., Yin X.L., Pang J.C., Zhu X.L., Poon W.S., Ng H.K. (2004). Detection of oncogene amplifications in medulloblastomas by comparative genomic hybridization and array-based comparative genomic hybridization. J. Neurosurg..

[57] Bean J., Brennan C., Shih J.Y., Riely G., Viale A., Wang L., Chitale D., Motoi N., Szoke J., Broderick S. (2007). Met amplification occurs with or without t790m mutations in egfr mutant lung tumors with acquired resistance to gefitinib or erlotinib. Proc. Natl. Acad. Sci. USA.

[58] Di Renzo M.F., Olivero M., Ferro S., Prat M., Bongarzone I., Pilotti S., Belfiore A., Costantino A., Vigneri R., Pierotti M.A. (1992). Overexpression of the c-met/hgf receptor gene in human thyroid carcinomas. Oncogene.

[59] Hiscox S.E., Hallett M.B., Puntis M.C., Nakamura T., Jiang W.G. (1997). Expression of the hgf/sf receptor, c-met, and its ligand in human colorectal cancers. Cancer Investig..

[60] Furukawa T., Duguid W.P., Kobari M., Matsuno S., Tsao M.S. (1995). Hepatocyte growth factor and met receptor expression in human pancreatic carcinogenesis. Am. J. Pathol..

[61] Di Renzo M.F., Olivero M., Katsaros D., Crepaldi T., Gaglia P., Zola P., Sismondi P., Comoglio P.M. (1994). Overexpression of the met/hgf receptor in ovarian cancer. Int. J. Cancer.

[62] Lengyel E., Prechtel D., Resau J.H., Gauger K., Welk A., Lindemann K., Salanti G., Richter T., Knudsen B., Vande Woude G.F. (2005). C-met overexpression in node-positive breast cancer identifies patients with poor clinical outcome independent of her2/neu. Int. J. Cancer.

[63] Schmidt L., Junker K., Nakaigawa N., Kinjerski T., Weirich G., Miller M., Lubensky I., Neumann H.P., Brauch H., Decker J. (1999). Novel mutations of the met proto-oncogene in papillary renal carcinomas. Oncogene.

[64] Pollak M. (2012). The insulin and insulin-like growth factor receptor family in neoplasia: An update. Nat. Rev. Cancer.

[65] Pollak M. (2008). Insulin and insulin-like growth factor signalling in neoplasia. Nat. Rev. Cancer.

[66] Murakami M.S., Rosen O.M. (1991). The role of insulin receptor autophosphorylation in signal transduction. J. Biol. Chem..

[67] Engelman J.A. (2009). Targeting PI3K signalling in cancer: Opportunities, challenges and limitations. Nat. Rev. Cancer.

[68] Ando A., Yonezawa K., Gout I., Nakata T., Ueda H., Hara K., Kitamura Y., Noda Y., Takenawa T., Hirokawa N. (1994). A complex of grb2-dynamin binds to tyrosine-phosphorylated insulin receptor substrate-1 after insulin treatment. EMBO J..

[69] Gan H.K., Burgess A.W., Clayton A.H., Scott A.M. (2012). Targeting of a conformationally exposed, tumor-specific epitope of EGFR as a strategy for cancer therapy. Cancer Res..

[70] Seshacharyulu P., Ponnusamy M.P., Haridas D., Jain M., Ganti A.K., Batra S.K. (2012). Targeting the EGFR signaling pathway in cancer therapy. Expert Opin. Ther. Targets.

[71] Sooro M.A., Zhang N., Zhang P. (2018). Targeting egfr-mediated autophagy as a potential strategy for cancer therapy. Int. J. Cancer.

[72] Yamaoka T., Ohba M., Ohmori T. (2017). Molecular-targeted therapies for epidermal growth factor receptor and its resistance mechanisms. Int. J. Mol. Sci..

[73] Johnston J.B., Navaratnam S., Pitz M.W., Maniate J.M., Wiechec E., Baust H., Gingerich J., Skliris G.P., Murphy L.C., Los M. (2006). Targeting the EGFR pathway for cancer therapy. Curr. Med. Chem..

[74] Metro G., Finocchiaro G., Cappuzzo F. (2006). Anti-cancer therapy with egfr inhibitors: Factors of prognostic and predictive significance. Ann. Oncol..

[75] Giaccone G., Gonzalez-Larriba J.L., van Oosterom A.T., Alfonso R., Smit E.F., Martens M., Peters G.J., van der Vijgh W.J., Smith R., Averbuch S. (2004). Combination therapy with gefitinib, an epidermal growth factor receptor tyrosine kinase inhibitor, gemcitabine and cisplatin in patients with advanced solid tumors. Ann. Oncol..

[76] Petrelli F., Borgonovo K., Cabiddu M., Ghilardi M., Barni S. (2011). Cetuximab and panitumumab in kras wild-type colorectal cancer: A meta-analysis. Int. J. Colorectal Dis..

[77] Rocha-Lima C.M., Soares H.P., Raez L.E., Singal R. (2007). Egfr targeting of solid tumors. Cancer Control.

[78] Tomasello C., Baldessari C., Napolitano M., Orsi G., Grizzi G., Bertolini F., Barbieri F., Cascinu S. (2018). Resistance to egfr inhibitors in non-small cell lung cancer: Clinical management and future perspectives. Crit. Rev. Oncol. Hematol..

[79] Ciardiello F., Tortora G. (2008). Egfr antagonists in cancer treatment. N. Engl. J. Med..

[80] Nan X., Xie C., Yu X., Liu J. (2017). Egfr tki as first-line treatment for patients with advanced egfr mutation-positive non-small-cell lung cancer. Oncotarget.

[81] Li J., Yan H. (2018). Skin toxicity with anti-egfr monoclonal antibody in cancer patients: A meta-analysis of 65 randomized controlled trials. Cancer Chemother. Pharmacol..

[82] Gorden K.J., Mesbah P., Kolesar J.M. (2012). Egfr inhibitors as first-line therapy in egfr mutation-positive patients with nsclc. J. Oncol. Pharm. Pract..

[83] Sandler A.B. (2006). Nondermatologic adverse events associated with anti-egfr therapy. Oncology.

[84] Vogel W.H., Jennifer P. (2016). Management strategies for adverse events associated with egfr tkis in non-small cell lung cancer. J. Adv. Pract. Oncol..

[85] Diaz-Serrano A., Gella P., Jimenez E., Zugazagoitia J., Paz-Ares Rodriguez L. (2018). Targeting egfr in lung cancer: Current standards and developments. Drugs.

[86] Sim E.H., Yang I.A., Wood-Baker R., Bowman R.V., Fong K.M. (2018). Gefitinib for advanced non-small cell lung cancer. Cochrane Database Syst. Rev..

[87] Landi L., Cappuzzo F. (2015). Experience with erlotinib in the treatment of non-small cell lung cancer. Ther. Adv. Respir. Dis..

[88] Genova C., Rijavec E., Barletta G., Burrafato G., Biello F., Dal Bello M.G., Coco S., Truini A., Alama A., Boccardo F. (2014). Afatinib for the treatment of advanced non-small-cell lung cancer. Expert Opin. Pharmacother..

[89] Jain P., Khanal R., Sharma A., Yan F., Sharma N. (2014). Afatinib and lung cancer. Expert Rev. Anticancer Ther..

[90] Keating G.M. (2016). Afatinib: A review in advanced non-small cell lung cancer. Targeted Oncol..

[91] Wirth S.M. (2015). Afatinib in non-small cell lung cancer. J. Adv. Pract. Oncol..

[92] Brzezniak C., Carter C.A., Giaccone G. (2013). Dacomitinib, a new therapy for the treatment of non-small cell lung cancer. Expert Opin. Pharmacother..

[93] Denis M.G., Vallee A., Theoleyre S. (2015). Egfr t790m resistance mutation in non small-cell lung carcinoma. Clin. Chim. Acta Int. J. Clin. Chem..

[94] Lim S.M., Syn N.L., Cho B.C., Soo R.A. (2018). Acquired resistance to egfr targeted therapy in non-small cell lung cancer: Mechanisms and therapeutic strategies. Cancer Treat. Rev..

[95] Liu Q., Yu S., Zhao W., Qin S., Chu Q., Wu K. (2018). Egfr-tkis resistance via egfr-independent signaling pathways. Mol. Cancer.

[96] Soejima K., Yasuda H., Hirano T. (2017). Osimertinib for egfr t790m mutation-positive non-small cell lung cancer. Expert Rev. Clin. Pharmacol..

[97] Wang S., Cang S., Liu D. (2016). Third-generation inhibitors targeting egfr t790m mutation in advanced non-small cell lung cancer. J. Hematol. Oncol..

[98] Wang Y., Guo Z., Li Y., Zhou Q. (2016). Development of epidermal growth factor receptor tyrosine kinase inhibitors against egfr t790m. Mutation in non small-cell lung carcinoma. Open Med..

[99] Akamatsu H., Katakami N., Okamoto I., Kato T., Kim Y.H., Imamura F., Shinkai M., Hodge R.A., Uchida H., Hida T. (2018). Osimertinib in japanese patients with egfr t790m mutation-positive advanced non-small-cell lung cancer: Aura3 trial. Cancer Sci..

[100] Soria J.C., Ohe Y., Vansteenkiste J., Reungwetwattana T., Chewaskulyong B., Lee K.H., Dechaphunkul A., Imamura F., Nogami N., Kurata T. (2018). Osimertinib in untreated egfr-mutated advanced non-small-cell lung cancer. N. Engl. J. Med..

[101] Lin J.Z., Ma S.K., Wu S.X., Yu S.H., Li X.Y. (2018). A network meta-analysis of nonsmall-cell lung cancer patients with an activating egfr mutation: Should osimertinib be the first-line treatment?. Medicine.

[102] White I.R. (2015). Network meta-analysis. Stata J..

[103] Wu Y.L., Zhou C., Hu C.P., Feng J., Lu S., Huang Y., Li W., Hou M., Shi J.H., Lee K.Y. (2014). Afatinib versus cisplatin plus gemcitabine for first-line treatment of asian patients with advanced non-small-cell lung cancer harbouring egfr mutations (lux-lung 6): An open-label, randomised phase 3 trial. Lancet Oncol..

[104] Zhou C., Wu Y.L., Chen G., Feng J., Liu X.Q., Wang C., Zhang S., Wang J., Zhou S., Ren S. (2011). Erlotinib versus chemotherapy as first-line treatment for patients with advanced egfr mutation-positive non-small-cell lung cancer (optimal, ctong-0802): A multicentre, open-label, randomised, phase 3 study. Lancet Oncol..

[105] Mitsudomi T., Morita S., Yatabe Y., Negoro S., Okamoto I., Tsurutani J., Seto T., Satouchi M., Tada H., Hirashima T. (2010). Gefitinib versus cisplatin plus docetaxel in patients with non-small-cell lung cancer harbouring mutations of the epidermal growth factor receptor (wjtog3405): An open label, randomised phase 3 trial. Lancet Oncol..

[106] Alorabi M., Shonka N.A., Ganti A.K. (2016). Egfr monoclonal antibodies in locally advanced head and neck squamous cell carcinoma: What is their current role?. Crit. Rev. Oncol. Hematol..

[107] van Helden E.J., Menke-van der Houven van Oordt C.W., Heymans M.W., Ket J.C.F., van den Oord R., Verheul H.M.W. (2017). Optimal use of anti-egfr monoclonal antibodies for patients with advanced colorectal cancer: A meta-analysis. Cancer Metastasis Rev..

[108] Vermorken J.B., Mesia R., Rivera F., Remenar E., Kawecki A., Rottey S., Erfan J., Zabolotnyy D., Kienzer H.R., Cupissol D. (2008). Platinum-based chemotherapy plus cetuximab in head and neck cancer. N. Engl. J. Med..

[109] Douillard J.Y., Oliner K.S., Siena S., Tabernero J., Burkes R., Barugel M., Humblet Y., Bodoky G., Cunningham D., Jassem J. (2013). Panitumumab-folfox4 treatment and ras mutations in colorectal cancer. N. Engl. J. Med..

[110] Huang L., Fu L. (2015). Mechanisms of resistance to egfr tyrosine kinase inhibitors. Acta Pharm. Sin. B.

[111] Morgillo F., Della Corte C.M., Fasano M., Ciardiello F. (2016). Mechanisms of resistance to egfr-targeted drugs: Lung cancer. ESMO Open.

[112] Barnes T.A., O’Kane G.M., Vincent M.D., Leighl N.B. (2017). Third-generation tyrosine kinase inhibitors targeting epidermal growth factor receptor mutations in non-small cell lung cancer. Front. Oncol..

[113] Russo A., Franchina T., Ricciardi G.R.R., Smiroldo V., Picciotto M., Zanghi M., Rolfo C., Adamo V. (2017). Third generation egfr tkis in egfr-mutated nsclc: Where are we now and where are we going. Crit. Rev. Oncol. Hematol..

[114] Tan C.S., Kumarakulasinghe N.B., Huang Y.Q., Ang Y.L.E., Choo J.R., Goh B.C., Soo R.A. (2018). Third generation egfr tkis: Current data and future directions. Mol. Cancer.

[115] Mok T.S., Wu Y.L., Ahn M.J., Garassino M.C., Kim H.R., Ramalingam S.S., Shepherd F.A., He Y., Akamatsu H., Theelen W.S. (2017). Osimertinib or platinum-pemetrexed in egfr t790m-positive lung cancer. N. Engl. J. Med..

[116] Lu X., Yu L., Zhang Z., Ren X., Smaill J.B., Ding K. (2018). Targeting EGFR(l858r/t790m) and EGFR(l858r/t790m/c797s) resistance mutations in NSCLC: Current developments in medicinal chemistry. Med. Res. Rev..

[117] Wu J.Y., Shih J.Y. (2016). Effectiveness of tyrosine kinase inhibitors on uncommon e709x epidermal growth factor receptor mutations in non-small-cell lung cancer. OncoTargets Ther..

[118] Kobayashi Y., Mitsudomi T. (2016). Not all epidermal growth factor receptor mutations in lung cancer are created equal: Perspectives for individualized treatment strategy. Cancer Sci..

[119] Guo G., Narayan R.N., Horton L., Patel T.R., Habib A.A. (2017). The role of EGFR-met interactions in the pathogenesis of glioblastoma and resistance to treatment. Current Cancer Drug Targets.

[120] Agwa E.S., Ma P.C. (2014). Targeting the met receptor tyrosine kinase in non-small cell lung cancer: Emerging role of tivantinib. Cancer Manag. Res..

[121] Abdelaziz A., Vaishampayan U. (2017). Cabozantinib for the treatment of kidney cancer. Expert Rev. Anticancer Ther..

[122] Yamaoka T., Ohmori T., Ohba M., Arata S., Kishino Y., Murata Y., Kusumoto S., Ishida H., Shirai T., Hirose T. (2016). Acquired resistance mechanisms to combination met-TKI/EGFR-TKI exposure in met-amplified EGFR-TKI-resistant lung adenocarcinoma harboring an activating EGFR mutation. Mol. Cancer Ther..

[123] Yamaoka T., Ohba M., Arata S., Ohmori T. (2017). Establishing dual resistance to EGFR-TKI and met-TKI in lung adenocarcinoma cells in vitro with a 2-step dose-escalation procedure. J. Vis. Exp..

[124] Yamaoka T., Ohmori T., Ohba M., Arata S., Murata Y., Kusumoto S., Ando K., Ishida H., Ohnishi T., Sasaki Y. (2017). Distinct afatinib resistance mechanisms identified in lung adenocarcinoma harboring an EGFR mutation. Mol. Cancer Res..

[125] Ando K., Ohmori T., Inoue F., Kadofuku T., Hosaka T., Ishida H., Shirai T., Okuda K., Hirose T., Horichi N. (2005). Enhancement of sensitivity to tumor necrosis factor alpha in non-small cell lung cancer cells with acquired resistance to GEFITINIB. Clin. Cancer Res..

[126] Sequist L.V., Waltman B.A., Dias-Santagata D., Digumarthy S., Turke A.B., Fidias P., Bergethon K., Shaw A.T., Gettinger S., Cosper A.K. (2011). Genotypic and histological evolution of lung cancers acquiring resistance to EGFR inhibitors. Sci. Transl. Med..

[127] Thiery J.P. (2003). Epithelial-mesenchymal transitions in development and pathologies. Curr. Opin. Cell Biol..

[128] Zhang Z., Lee J.C., Lin L., Olivas V., Au V., LaFramboise T., Abdel-Rahman M., Wang X., Levine A.D., Rho J.K. (2012). Activation of the AXL kinase causes resistance to EGFR-targeted therapy in lung cancer. Nat. Genet..

[129] Bivona T.G., Hieronymus H., Parker J., Chang K., Taron M., Rosell R., Moonsamy P., Dahlman K., Miller V.A., Costa C. (2011). Fas and NF-κB signalling modulate dependence of lung cancers on mutant EGFR. Nature.

[130] Stewart E.L., Tan S.Z., Liu G., Tsao M.S. (2015). Known and putative mechanisms of resistance to egfr targeted therapies in NSCLC patients with EGFR mutations—A review. Transl. Lung Cancer Res..

[131] Amado R.G., Wolf M., Peeters M., Van Cutsem E., Siena S., Freeman D.J., Juan T., Sikorski R., Suggs S., Radinsky R. (2008). Wild-type kras is required for panitumumab efficacy in patients with metastatic colorectal cancer. J. Clin. Oncol..

[132] Karapetis C.S., Khambata-Ford S., Jonker D.J., O’Callaghan C.J., Tu D., Tebbutt N.C., Simes R.J., Chalchal H., Shapiro J.D., Robitaille S. (2008). K-ras mutations and benefit from cetuximab in advanced colorectal cancer. N. Engl. J. Med..

[133] Weickhardt A.J., Price T.J., Chong G., Gebski V., Pavlakis N., Johns T.G., Azad A., Skrinos E., Fluck K., Dobrovic A. (2012). Dual targeting of the epidermal growth factor receptor using the combination of cetuximab and erlotinib: Preclinical evaluation and results of the phase ii dux study in chemotherapy-refractory, advanced colorectal cancer. J. Clin. Oncol..

[134] Van Cutsem E., Eng C., Nowara E., Swieboda-Sadlej A., Tebbutt N.C., Mitchell E., Davidenko I., Stephenson J., Elez E., Prenen H. (2014). Randomized phase IB/II trial of rilotumumab or ganitumab with panitumumab versus panitumumab alone in patients with wild-type KRAS metastatic colorectal cancer. Clin. Cancer Res..

[135] Russo M., Siravegna G., Blaszkowsky L.S., Corti G., Crisafulli G., Ahronian L.G., Mussolin B., Kwak E.L., Buscarino M., Lazzari L. (2016). Tumor heterogeneity and lesion-specific response to targeted therapy in colorectal cancer. Cancer Discov..

[136] Lewis G.D., Figari I., Fendly B., Wong W.L., Carter P., Gorman C., Shepard H.M. (1993). Differential responses of human tumor cell lines to anti-p185her2 monoclonal antibodies. Cancer Immunol. Immunother..

[137] Nagata Y., Lan K.H., Zhou X., Tan M., Esteva F.J., Sahin A.A., Klos K.S., Li P., Monia B.P., Nguyen N.T. (2004). Pten activation contributes to tumor inhibition by trastuzumab, and loss of pten predicts trastuzumab resistance in patients. Cancer Cell.

[138] Moasser M.M. (2007). Targeting the function of the her2 oncogene in human cancer therapeutics. Oncogene.

[139] Shi Y., Fan X., Meng W., Deng H., Zhang N., An Z. (2014). Engagement of immune effector cells by trastuzumab induces her2/erbb2 downregulation in cancer cells through stat1 activation. Breast Cancer Res..

[140] Gradishar W.J. (2013). Emerging approaches for treating her2-positive metastatic breast cancer beyond trastuzumab. Ann. Oncol..

[141] Rusnak D.W., Lackey K., Affleck K., Wood E.R., Alligood K.J., Rhodes N., Keith B.R., Murray D.M., Knight W.B., Mullin R.J. (2001). The effects of the novel, reversible epidermal growth factor receptor/erbb-2 tyrosine kinase inhibitor, gw2016, on the growth of human normal and tumor-derived cell lines in vitro and in vivo. Mol. Cancer Ther..

[142] Rabindran S.K., Discafani C.M., Rosfjord E.C., Baxter M., Floyd M.B., Golas J., Hallett W.A., Johnson B.D., Nilakantan R., Overbeek E. (2004). Antitumor activity of hki-272, an orally active, irreversible inhibitor of the her-2 tyrosine kinase. Cancer Res..

[143] Christianson T.A., Doherty J.K., Lin Y.J., Ramsey E.E., Holmes R., Keenan E.J., Clinton G.M. (1998). Nh2-terminally truncated her-2/neu protein: Relationship with shedding of the extracellular domain and with prognostic factors in breast cancer. Cancer Res..

[144] Molina M.A., Saez R., Ramsey E.E., Garcia-Barchino M.J., Rojo F., Evans A.J., Albanell J., Keenan E.J., Lluch A., Garcia-Conde J. (2002). Nh(2)-terminal truncated her-2 protein but not full-length receptor is associated with nodal metastasis in human breast cancer. Clin. Cancer Res..

[145] Scaltriti M., Rojo F., Ocana A., Anido J., Guzman M., Cortes J., Di Cosimo S., Matias-Guiu X., Ramon y Cajal S., Arribas J. (2007). Expression of p95her2, a truncated form of the her2 receptor, and response to anti-her2 therapies in breast cancer. J. Natl. Cancer Inst..

[146] Stephens P., Hunter C., Bignell G., Edkins S., Davies H., Teague J., Stevens C., O’Meara S., Smith R., Parker A. (2004). Lung cancer: Intragenic erbb2 kinase mutations in tumours. Nature.

[147] Cohen E.E., Lingen M.W., Martin L.E., Harris P.L., Brannigan B.W., Haserlat S.M., Okimoto R.A., Sgroi D.C., Dahiya S., Muir B. (2005). Response of some head and neck cancers to epidermal growth factor receptor tyrosine kinase inhibitors may be linked to mutation of erbb2 rather than EGFR. Clin. Cancer Res..

[148] Lee J.W., Soung Y.H., Seo S.H., Kim S.Y., Park C.H., Wang Y.P., Park K., Nam S.W., Park W.S., Kim S.H. (2006). Somatic mutations of erbb2 kinase domain in gastric, colorectal, and breast carcinomas. Clin. Cancer Res..

[149] Trowe T., Boukouvala S., Calkins K., Cutler R.E., Fong R., Funke R., Gendreau S.B., Kim Y.D., Miller N., Woolfrey J.R. (2008). Exel-7647 inhibits mutant forms of ERBB2 associated with lapatinib resistance and neoplastic transformation. Clin. Cancer Res..

[150] Hanker A.B., Brewer M.R., Sheehan J.H., Koch J.P., Sliwoski G.R., Nagy R., Lanman R., Berger M.F., Hyman D.M., Solit D.B. (2017). An acquired HER2(T798I) gatekeeper mutation induces resistance to neratinib in a patient with her2 mutant-driven breast cancer. Cancer Discov..

[151] Mittendorf E.A., Wu Y., Scaltriti M., Meric-Bernstam F., Hunt K.K., Dawood S., Esteva F.J., Buzdar A.U., Chen H., Eksambi S. (2009). Loss of her2 amplification following trastuzumab-based neoadjuvant systemic therapy and survival outcomes. Clin. Cancer Res..

[152] Garrett J.T., Olivares M.G., Rinehart C., Granja-Ingram N.D., Sanchez V., Chakrabarty A., Dave B., Cook R.S., Pao W., McKinely E. (2011). Transcriptional and posttranslational up-regulation of her3 (erbb3) compensates for inhibition of the her2 tyrosine kinase. Proc. Natl. Acad. Sci. USA.

[153] Wehrman T.S., Raab W.J., Casipit C.L., Doyonnas R., Pomerantz J.H., Blau H.M. (2006). A system for quantifying dynamic protein interactions defines a role for herceptin in modulating erbb2 interactions. Proc. Natl. Acad. Sci. USA.

[154] Shattuck D.L., Miller J.K., Carraway K.L., Sweeney C. (2008). Met receptor contributes to trastuzumab resistance of her2-overexpressing breast cancer cells. Cancer Res..

[155] Chen C.T., Kim H., Liska D., Gao S., Christensen J.G., Weiser M.R. (2012). Met activation mediates resistance to lapatinib inhibition of her2-amplified gastric cancer cells. Mol. Cancer Ther..

[156] Liu L., Greger J., Shi H., Liu Y., Greshock J., Annan R., Halsey W., Sathe G.M., Martin A.M., Gilmer T.M. (2009). Novel mechanism of lapatinib resistance in her2-positive breast tumor cells: Activation of AXL. Cancer Res..

[157] Harris L.N., You F., Schnitt S.J., Witkiewicz A., Lu X., Sgroi D., Ryan P.D., Come S.E., Burstein H.J., Lesnikoski B.A. (2007). Predictors of resistance to preoperative trastuzumab and vinorelbine for her2-positive early breast cancer. Clin. Cancer Res..

[158] Arteaga C.L. (2007). Her3 and mutant EGFR meet met. Nat. Med..

[159] Bae S.Y., Hong J.Y., Lee H.J., Park H.J., Lee S.K. (2015). Targeting the degradation of axl receptor tyrosine kinase to overcome resistance in gefitinib-resistant non-small cell lung cancer. Oncotarget.

[160] Jameson M.J., Beckler A.D., Taniguchi L.E., Allak A., Vanwagner L.B., Lee N.G., Thomsen W.C., Hubbard M.A., Thomas C.Y. (2011). Activation of the insulin-like growth factor-1 receptor induces resistance to epidermal growth factor receptor antagonism in head and neck squamous carcinoma cells. Mol. Cancer Ther..

[161] Jimenez C., Jones D.R., Rodriguez-Viciana P., Gonzalez-Garcia A., Leonardo E., Wennstrom S., von Kobbe C., Toran J.L., L R.B., Calvo V. (1998). Identification and characterization of a new oncogene derived from the regulatory subunit of phosphoinositide 3-kinase. EMBO J..

[162] Philp A.J., Campbell I.G., Leet C., Vincan E., Rockman S.P., Whitehead R.H., Thomas R.J., Phillips W.A. (2001). The phosphatidylinositol 3’-kinase p85alpha gene is an oncogene in human ovarian and colon tumors. Cancer Res..

[163] Chen Z., Trotman L.C., Shaffer D., Lin H.K., Dotan Z.A., Niki M., Koutcher J.A., Scher H.I., Ludwig T., Gerald W. (2005). Crucial role of p53-dependent cellular senescence in suppression of pten-deficient tumorigenesis. Nature.

[164] Elster N., Cremona M., Morgan C., Toomey S., Carr A., O’Grady A., Hennessy B.T., Eustace A.J. (2015). A preclinical evaluation of the pi3k alpha/delta dominant inhibitor bay 80-6946 in her2-positive breast cancer models with acquired resistance to the her2-targeted therapies trastuzumab and lapatinib. Breast Cancer Res. Treat..

[165] Thomas S.M., Brugge J.S. (1997). Cellular functions regulated by SRC family kinases. Annu. Rev. Cell Dev. Biology.

[166] Dehm S.M., Bonham K. (2004). Src gene expression in human cancer: The role of transcriptional activation. Biochem. Cell Biol..

[167] Rexer B.N., Ham A.J., Rinehart C., Hill S., Granja-Ingram Nde M., Gonzalez-Angulo A.M., Mills G.B., Dave B., Chang J.C., Liebler D.C. (2011). Phosphoproteomic mass spectrometry profiling links src family kinases to escape from her2 tyrosine kinase inhibition. Oncogene.

[168] Warmerdam P.A., van de Winkel J.G., Vlug A., Westerdaal N.A., Capel P.J. (1991). A single amino acid in the second ig-like domain of the human fc gamma receptor ii is critical for human igg2 binding. J. Immunol..

[169] Koene H.R., Kleijer M., Algra J., Roos D., von dem Borne A.E., de Haas M. (1997). Fc gammariiia-158v/f polymorphism influences the binding of igg by natural killer cell fc gammariiia, independently of the fc gammariiia-48l/r/h phenotype. Blood.

[170] Musolino A., Naldi N., Bortesi B., Pezzuolo D., Capelletti M., Missale G., Laccabue D., Zerbini A., Camisa R., Bisagni G. (2008). Immunoglobulin g fragment c receptor polymorphisms and clinical efficacy of trastuzumab-based therapy in patients with her-2/neu-positive metastatic breast cancer. J. Clin. Oncol..

[171] Roca L., Dieras V., Roche H., Lappartient E., Kerbrat P., Cany L., Chieze S., Canon J.L., Spielmann M., Penault-Llorca F. (2013). Correlation of her2, fcgr2a, and fcgr3a gene polymorphisms with trastuzumab related cardiac toxicity and efficacy in a subgroup of patients from unicancer-pacs 04 trial. Breast Cancer Res. Treat..

[172] Shimizu C., Mogushi K., Morioka M.S., Yamamoto H., Tamura K., Fujiwara Y., Tanaka H. (2016). Fc-gamma receptor polymorphism and gene expression of peripheral blood mononuclear cells in patients with her2-positive metastatic breast cancer receiving single-agent trastuzumab. Breast Cancer.

[173] Morris S.W., Kirstein M.N., Valentine M.B., Dittmer K.G., Shapiro D.N., Saltman D.L., Look A.T. (1994). Fusion of a kinase gene, alk, to a nucleolar protein gene, npm, in non-hodgkin’s lymphoma. Science.

[174] Reshetnyak A.V., Mohanty J., Tome F., Puleo D.E., Plotnikov A.N., Ahmed M., Kaur N., Poliakov A., Cinnaiyan A.M., Lax I. (2018). Identification of a biologically active fragment of ALK and LTK-ligand 2 (augmentor-alpha). Proc. Natl. Acad. Sci. USA.

[175] Reshetnyak A.V., Murray P.B., Shi X., Mo E.S., Mohanty J., Tome F., Bai H., Gunel M., Lax I., Schlessinger J. (2015). Augmentor alpha and beta (fam150) are ligands of the receptor tyrosine kinases alk and ltk: Hierarchy and specificity of ligand-receptor interactions. Proc. Natl. Acad. Sci. USA.

[176] Shaw A.T., Yeap B.Y., Mino-Kenudson M., Digumarthy S.R., Costa D.B., Heist R.S., Solomon B., Stubbs H., Admane S., McDermott U. (2009). Clinical features and outcome of patients with non-small-cell lung cancer who harbor eml4-alk. J. Clin. Oncol..

[177] Solomon B.J., Mok T., Kim D.W., Wu Y.L., Nakagawa K., Mekhail T., Felip E., Cappuzzo F., Paolini J., Usari T. (2014). First-line crizotinib versus chemotherapy in alk-positive lung cancer. N. Engl. J. Med..

[178] Katayama R., Shaw A.T., Khan T.M., Mino-Kenudson M., Solomon B.J., Halmos B., Jessop N.A., Wain J.C., Yeo A.T., Benes C. (2012). Mechanisms of acquired crizotinib resistance in alk-rearranged lung cancers. Sci. Transl. Med..

[179] Crystal A.S., Shaw A.T., Sequist L.V., Friboulet L., Niederst M.J., Lockerman E.L., Frias R.L., Gainor J.F., Amzallag A., Greninger P. (2014). Patient-derived models of acquired resistance can identify effective drug combinations for cancer. Science.

[180] Lovly C.M., McDonald N.T., Chen H., Ortiz-Cuaran S., Heukamp L.C., Yan Y., Florin A., Ozretic L., Lim D., Wang L. (2014). Rationale for co-targeting IGF-1R and ALK in ALK fusion-positive lung cancer. Nat. Med..

[181] Cha Y.J., Cho B.C., Kim H.R., Lee H.J., Shim H.S. (2016). A case of alk-rearranged adenocarcinoma with small cell carcinoma-like transformation and resistance to crizotinib. J. Thorac. Oncol..

[182] Gainor J.F., Dardaei L., Yoda S., Friboulet L., Leshchiner I., Katayama R., Dagogo-Jack I., Gadgeel S., Schultz K., Singh M. (2016). Molecular mechanisms of resistance to first- and second-generation alk inhibitors in alk-rearranged lung cancer. Cancer Discov..

[183] Kim H.R., Kim W.S., Choi Y.J., Choi C.M., Rho J.K., Lee J.C. (2013). Epithelial-mesenchymal transition leads to crizotinib resistance in h2228 lung cancer cells with eml4-alk translocation. Mol. Oncol..

[184] Dardaei L., Wang H.Q., Singh M., Fordjour P., Shaw K.X., Yoda S., Kerr G., Yu K., Liang J., Cao Y. (2018). Shp2 inhibition restores sensitivity in alk-rearranged non-small-cell lung cancer resistant to alk inhibitors. Nat. Med..

[185] Doebele R.C., Pilling A.B., Aisner D.L., Kutateladze T.G., Le A.T., Weickhardt A.J., Kondo K.L., Linderman D.J., Heasley L.E., Franklin W.A. (2012). Mechanisms of resistance to crizotinib in patients with alk gene rearranged non-small cell lung cancer. Clin. Cancer Res..

[186] Lin J.J., Riely G.J., Shaw A.T. (2017). Targeting ALK: Precision medicine takes on drug resistance. Cancer Discov..

[187] Katayama R., Sakashita T., Yanagitani N., Ninomiya H., Horiike A., Friboulet L., Gainor J.F., Motoi N., Dobashi A., Sakata S. (2016). P-glycoprotein mediates ceritinib resistance in anaplastic lymphoma kinase-rearranged non-small cell lung cancer. EBioMedicine.

[188] Soria J.C., Tan D.S.W., Chiari R., Wu Y.L., Paz-Ares L., Wolf J., Geater S.L., Orlov S., Cortinovis D., Yu C.J. (2017). First-line ceritinib versus platinum-based chemotherapy in advanced alk-rearranged non-small-cell lung cancer (ascend-4): A randomised, open-label, phase 3 study. Lancet.

[189] Peters S., Camidge D.R., Shaw A.T., Gadgeel S., Ahn J.S., Kim D.W., Ou S.I., Perol M., Dziadziuszko R., Rosell R. (2017). Alectinib versus crizotinib in untreated alk-positive non-small-cell lung cancer. N. Engl. J. Med..

[190] FDA Highlights of Prescribing Information. https://www.Accessdata.Fda.Gov/drugsatfda_docs/label/2017/208434s003lbl.Pdf.

[191] Hida T., Nokihara H., Kondo M., Kim Y.H., Azuma K., Seto T., Takiguchi Y., Nishio M., Yoshioka H., Imamura F. (2017). Alectinib versus crizotinib in patients with alk-positive non-small-cell lung cancer (j-alex): An open-label, randomised phase 3 trial. Lancet.

[192] Kim D.W., Tiseo M., Ahn M.J., Reckamp K.L., Hansen K.H., Kim S.W., Huber R.M., West H.L., Groen H.J.M., Hochmair M.J. (2017). Brigatinib in patients with crizotinib-refractory anaplastic lymphoma kinase-positive non-small-cell lung cancer: A randomized, multicenter phase ii trial. J. Clin. Oncol..

[193] Shaw A.T., Felip E., Bauer T.M., Besse B., Navarro A., Postel-Vinay S., Gainor J.F., Johnson M., Dietrich J., James L.P. (2017). Lorlatinib in non-small-cell lung cancer with alk or ros1 rearrangement: An international, multicentre, open-label, single-arm first-in-man phase 1 trial. Lancet Oncol..

[194] Zhao Y., Adjei A.A. (2015). Targeting angiogenesis in cancer therapy: Moving beyond vascular endothelial growth factor. Oncologist.

[195] Ilic I., Jankovic S., Ilic M. (2016). Bevacizumab combined with chemotherapy improves survival for patients with metastatic colorectal cancer: Evidence from meta analysis. PLoS ONE.

[196] Sandler A., Gray R., Perry M.C., Brahmer J., Schiller J.H., Dowlati A., Lilenbaum R., Johnson D.H. (2006). Paclitaxel-carboplatin alone or with bevacizumab for non-small-cell lung cancer. N. Engl. J. Med..

[197] Pujade-Lauraine E., Hilpert F., Weber B., Reuss A., Poveda A., Kristensen G., Sorio R., Vergote I., Witteveen P., Bamias A. (2014). Bevacizumab combined with chemotherapy for platinum-resistant recurrent ovarian cancer: The aurelia open-label randomized phase iii trial. J. Clin. Oncol..

[198] Wilke H., Muro K., Van Cutsem E., Oh S.C., Bodoky G., Shimada Y., Hironaka S., Sugimoto N., Lipatov O., Kim T.Y. (2014). Ramucirumab plus paclitaxel versus placebo plus paclitaxel in patients with previously treated advanced gastric or gastro-oesophageal junction adenocarcinoma (rainbow): A double-blind, randomised phase 3 trial. Lancet Oncol..

[199] Tabernero J., Takayuki Y., Cohn A.L. (2015). Ramucirumab versus placebo in combination with second-line folfiri in patients with metastatic colorectal carcinoma that progressed during or after first-line therapy with bevacizumab, oxaliplatin, and a fluoropyrimidine (raise): A randomised, double-blind, multicentre, phase 3 study. Lancet Oncol..

[200] Garon E.B., Ciuleanu T.E., Arrieta O., Prabhash K., Syrigos K.N., Goksel T., Park K., Gorbunova V., Kowalyszyn R.D., Pikiel J. (2014). Ramucirumab plus docetaxel versus placebo plus docetaxel for second-line treatment of stage iv non-small-cell lung cancer after disease progression on platinum-based therapy (revel): A multicentre, double-blind, randomised phase 3 trial. Lancet.

[201] Van Cutsem E., Tabernero J., Lakomy R., Prenen H., Prausová J., Macarulla T., Ruff P., van Hazel G.A., Moiseyenko V., Ferry D. (2012). Addition of aflibercept to fluorouracil, leucovorin, and irinotecan improves survival in a phase iii randomized trial in patients with metastatic colorectal cancer previously treated with an oxaliplatin-based regimen. J. Clin. Oncol..

[202] Casanovas O., Hicklin D.J., Bergers G., Hanahan D. (2005). Drug resistance by evasion of antiangiogenic targeting of vegf signaling in late-stage pancreatic islet tumors. Cancer Cell.

[203] Escudier B., Grünwald V., Ravaud A., Ou Y.C., Castellano D., Lin C.C., Gschwend J.E., Harzstark A., Beall S., Pirotta N. (2014). Phase ii results of dovitinib (tki258) in patients with metastatic renal cell cancer. Clin. Cancer Res..

[204] Motzer R.J., Porta C., Vogelzang N.J., Sternberg C.N., Szczylik C., Zolnierek J., Kollmannsberger C., Rha S.Y., Bjarnason G.A., Melichar B. (2014). Dovitinib versus sorafenib for third-line targeted treatment of patients with metastatic renal cell carcinoma: An open-label, randomised phase 3 trial. Lancet Oncol..

[205] Rigamonti N., Kadioglu E., Keklikoglou I., Wyser Rmili C., Leow C.C., De Palma M. (2014). Role of angiopoietin-2 in adaptive tumor resistance to vegf signaling blockade. Cell Rep..

[206] Biel N.M., Siemann D.W. (2016). Targeting the angiopoietin-2/tie-2 axis in conjunction with vegf signal interference. Cancer Lett.

[207] Mamer S.B., Chen S., Weddell J.C., Palasz A., Wittenkeller A., Kumar M., Imoukhuede P.I. (2017). Discovery of high-affinity pdgf-vegfr interactions: Redefining rtk dynamics. Sci. Rep..

[208] Lu K.V., Chang J.P., Parachoniak C.A., Pandika M.M., Aghi M.K., Meyronet D., Isachenko N., Fouse S.D., Phillips J.J., Cheresh D.A. (2012). Vegf inhibits tumor cell invasion and mesenchymal transition through a met/vegfr2 complex. Cancer Cell.

[209] Choueiri T.K., Escudier B., Powles T., Tannir N.M., Mainwaring P.N., Rini B.I., Hammers H.J., Donskov F., Roth B.J., Peltola K. (2016). Cabozantinib versus everolimus in advanced renal cell carcinoma (meteor): Final results from a randomised, open-label, phase 3 trial. Lancet Oncol..

[210] Sattler M., Reddy M.M., Hasina R., Gangadhar T., Salgia R. (2011). The role of the c-met pathway in lung cancer and the potential for targeted therapy. Ther. Adv. Med. Oncol..

[211] Catenacci D.V.T., Tebbutt N.C., Davidenko I., Murad A.M., Al-Batran S.E., Ilson D.H., Tjulandin S., Gotovkin E., Karaszewska B., Bondarenko I. (2017). Rilotumumab plus epirubicin, cisplatin, and capecitabine as first-line therapy in advanced met-positive gastric or gastro-oesophageal junction cancer (rilomet-1): A randomised, double-blind, placebo-controlled, phase 3 trial. Lancet Oncol..

[212] Spigel D.R., Edelman M.J., O’Byrne K., Paz-Ares L., Mocci S., Phan S., Shames D.S., Smith D., Yu W., Paton V.E. (2017). Results from the phase iii randomized trial of onartuzumab plus erlotinib versus erlotinib in previously treated stage iiib or iv non-small-cell lung cancer: Metlung. J. Clin. Oncol..

[213] Shah M.A., Bang Y.J., Lordick F., Alsina M., Chen M., Hack S.P., Bruey J.M., Smith D., McCaffery I., Shames D.S. (2017). Effect of fluorouracil, leucovorin, and oxaliplatin with or without onartuzumab in her2-negative, met-positive gastroesophageal adenocarcinoma: The metgastric randomized clinical trial. JAMA Oncol..

[214] Shaw A.T., Kim D.W., Nakagawa K., Seto T., Crino L., Ahn M.J., De Pas T., Besse B., Solomon B.J., Blackhall F. (2013). Crizotinib versus chemotherapy in advanced alk-positive lung cancer. N. Engl. J. Med..

[215] Abou-Alfa G.K., Meyer T., Cheng A.L., El-Khoueiry A.B., Rimassa L., Ryoo B.Y., Cicin I., Merle P., Chen Y., Park J.W. (2018). Cabozantinib in patients with advanced and progressing hepatocellular carcinoma. N. Engl. J. Med..

[216] Cella D., Escudier B., Tannir N.M., Powles T., Donskov F., Peltola K., Schmidinger M., Heng D.Y.C., Mainwaring P.N., Hammers H.J. (2018). Quality of life outcomes for cabozantinib versus everolimus in patients with metastatic renal cell carcinoma: Meteor phase iii randomized trial. J. Clin. Oncol..

[217] Smith M., De Bono J., Sternberg C., Le Moulec S., Oudard S., De Giorgi U., Krainer M., Bergman A., Hoelzer W., De Wit R. (2016). Phase iii study of cabozantinib in previously treated metastatic castration-resistant prostate cancer: Comet-1. J. Clin. Oncol..

[218] Elisei R., Schlumberger M.J., Muller S.P., Schoffski P., Brose M.S., Shah M.H., Licitra L., Jarzab B., Medvedev V., Kreissl M.C. (2013). Cabozantinib in progressive medullary thyroid cancer. J. Clin. Oncol..

[219] Cepero V., Sierra J.R., Corso S., Ghiso E., Casorzo L., Perera T., Comoglio P.M., Giordano S. (2010). Met and kras gene amplification mediates acquired resistance to met tyrosine kinase inhibitors. Cancer Res..

[220] Bahcall M., Sim T., Paweletz C.P., Patel J.D., Alden R.S., Kuang Y., Sacher A.G., Kim N.D., Lydon C.A., Awad M.M. (2016). Acquired metd1228v mutation and resistance to met inhibition in lung cancer. Cancer Discov..

[221] Martin V., Corso S., Comoglio P.M., Giordano S. (2014). Increase of met gene copy number confers resistance to a monovalent met antibody and establishes drug dependence. Mol. Oncol..

[222] Corso S., Ghiso E., Cepero V., Sierra J.R., Migliore C., Bertotti A., Trusolino L., Comoglio P.M., Giordano S. (2010). Activation of her family members in gastric carcinoma cells mediates resistance to met inhibition. Mol. Cancer.

[223] Migliore C., Morando E., Ghiso E., Anastasi S., Leoni V.P., Apicella M., Cora D., Sapino A., Pietrantonio F., De Braud F. (2018). Mir-205 mediates adaptive resistance to met inhibition via errfi1 targeting and raised egfr signaling. EMBO Mol. Med..

[224] Lipton J.H., Chuah C., Guerci-Bresler A., Rosti G., Simpson D., Assouline S., Etienne G., Nicolini F.E., le Coutre P., Clark R.E. (2016). Ponatinib versus imatinib for newly diagnosed chronic myeloid leukaemia: An international, randomised, open-label, phase 3 trial. Lancet Oncol..

[225] Paik P.K., Shen R., Berger M.F., Ferry D., Soria J.C., Mathewson A., Rooney C., Smith N.R., Cullberg M., Kilgour E. (2017). A phase ib open-label multicenter study of azd4547 in patients with advanced squamous cell lung cancers. Clin. Cancer Res..

[226] Nogova L., Sequist L.V., Perez Garcia J.M., Andre F., Delord J.P., Hidalgo M., Schellens J.H., Cassier P.A., Camidge D.R., Schuler M. (2017). Evaluation of bgj398, a fibroblast growth factor receptor 1-3 kinase inhibitor, in patients with advanced solid tumors harboring genetic alterations in fibroblast growth factor receptors: Results of a global phase i, dose-escalation and dose-expansion study. J. Clin. Oncol..

[227] Tabernero J., Bahleda R., Dienstmann R., Infante J.R., Mita A., Italiano A., Calvo E., Moreno V., Adamo B., Gazzah A. (2015). Phase i dose-escalation study of jnj-42756493, an oral pan-fibroblast growth factor receptor inhibitor, in patients with advanced solid tumors. J. Clin. Oncol..

[228] Helsten T., Schwaederle M., Kurzrock R. (2015). Fibroblast growth factor receptor signaling in hereditary and neoplastic disease: Biologic and clinical implications. Cancer Metastasis Rev..

[229] Chell V., Balmanno K., Little A.S., Wilson M., Andrews S., Blockley L., Hampson M., Gavine P.R., Cook S.J. (2013). Tumour cell responses to new fibroblast growth factor receptor tyrosine kinase inhibitors and identification of a gatekeeper mutation in fgfr3 as a mechanism of acquired resistance. Oncogene.

[230] Byron S.A., Chen H., Wortmann A., Loch D., Gartside M.G., Dehkhoda F., Blais S.P., Neubert T.A., Mohammadi M., Pollock P.M. (2013). The n550k/h mutations in fgfr2 confer differential resistance to pd173074, dovitinib, and ponatinib atp-competitive inhibitors. Neoplasia.

[231] Tan L., Wang J., Tanizaki J., Huang Z., Aref A.R., Rusan M., Zhu S.J., Zhang Y., Ercan D., Liao R.G. (2014). Development of covalent inhibitors that can overcome resistance to first-generation fgfr kinase inhibitors. Proc. Natl. Acad. Sci. USA.

[232] Porta R., Borea R., Coelho A., Khan S., Araujo A., Reclusa P., Franchina T., Van Der Steen N., Van Dam P., Ferri J. (2017). Fgfr a promising druggable target in cancer: Molecular biology and new drugs. Crit Rev Oncol Hematol.

[233] Sell C., Dumenil G., Deveaud C., Miura M., Coppola D., DeAngelis T., Rubin R., Efstratiadis A., Baserga R. (1994). Effect of a null mutation of the insulin-like growth factor i receptor gene on growth and transformation of mouse embryo fibroblasts. Mol. Cell. Biol..

[234] Zha J., O’Brien C., Savage H., Huw L.Y., Zhong F., Berry L., Lewis Phillips G.D., Luis E., Cavet G., Hu X. (2009). Molecular predictors of response to a humanized anti-insulin-like growth factor-i receptor monoclonal antibody in breast and colorectal cancer. Mol. Cancer Ther..

[235] Asmane I., Watkin E., Alberti L., Duc A., Marec-Berard P., Ray-Coquard I., Cassier P., Decouvelaere A.V., Ranchere D., Kurtz J.E. (2012). Insulin-like growth factor type 1 receptor (igf-1r) exclusive nuclear staining: A predictive biomarker for igf-1r monoclonal antibody (ab) therapy in sarcomas. Eur. J. Cancer.

[236] Gualberto A., Hixon M.L., Karp D.D., Li D., Green S., Dolled-Filhart M., Paz-Ares L.G., Novello S., Blakely J., Langer C.J. (2011). Pre-treatment levels of circulating free igf-1 identify nsclc patients who derive clinical benefit from figitumumab. Br. J. Cancer.

[237] Langer C.J., Novello S., Park K., Krzakowski M., Karp D.D., Mok T., Benner R.J., Scranton J.R., Olszanski A.J., Jassem J. (2014). Randomized, phase iii trial of first-line figitumumab in combination with paclitaxel and carboplatin versus paclitaxel and carboplatin alone in patients with advanced non-small-cell lung cancer. J. Clin. Oncol..

[238] Scagliotti G.V., Bondarenko I., Blackhall F., Barlesi F., Hsia T.C., Jassem J., Milanowski J., Popat S., Sanchez-Torres J.M., Novello S. (2015). Randomized, phase iii trial of figitumumab in combination with erlotinib versus erlotinib alone in patients with nonadenocarcinoma nonsmall-cell lung cancer. Ann. Oncol..

[239] Fuchs C.S., Azevedo S., Okusaka T., Van Laethem J.L., Lipton L.R., Riess H., Szczylik C., Moore M.J., Peeters M., Bodoky G. (2015). A phase 3 randomized, double-blind, placebo-controlled trial of ganitumab or placebo in combination with gemcitabine as first-line therapy for metastatic adenocarcinoma of the pancreas: The gamma trial. Ann. Oncol..

[240] Fassnacht M., Berruti A., Baudin E., Demeure M.J., Gilbert J., Haak H., Kroiss M., Quinn D.I., Hesseltine E., Ronchi C.L. (2015). Linsitinib (osi-906) versus placebo for patients with locally advanced or metastatic adrenocortical carcinoma: A double-blind, randomised, phase 3 study. Lancet Oncol..

[241] Leighl N.B., Rizvi N.A., de Lima L.G., Arpornwirat W., Rudin C.M., Chiappori A.A., Ahn M.J., Chow L.Q., Bazhenova L., Dechaphunkul A. (2017). Phase 2 study of erlotinib in combination with linsitinib (osi-906) or placebo in chemotherapy-naive patients with non-small-cell lung cancer and activating epidermal growth factor receptor mutations. Clin. Lung Cancer.

[242] Ulanet D.B., Ludwig D.L., Kahn C.R., Hanahan D. (2010). Insulin receptor functionally enhances multistage tumor progression and conveys intrinsic resistance to igf-1r targeted therapy. Proc. Natl. Acad. Sci. USA.

[243] Flaherty K.T., Hodi F.S., Fisher D.E. (2012). From genes to drugs: Targeted strategies for melanoma. Nat. Rev. Cancer.

[244] Kindler T., Breitenbuecher F., Marx A., Beck J., Hess G., Weinkauf B., Duyster J., Peschel C., Kirkpatrick C.J., Theobald M. (2004). Efficacy and safety of imatinib in adult patients with c-kit-positive acute myeloid leukemia. Blood.

[245] Pardanani A., Tefferi A. (2010). Systemic mastocytosis in adults: A review on prognosis and treatment based on 342 mayo clinic patients and current literature. Curr. Opin. Hematol..

[246] Gramza A.W., Corless C.L., Heinrich M.C. (2009). Resistance to tyrosine kinase inhibitors in gastrointestinal stromal tumors. Clin. Cancer Res..

[247] Demetri G.D., von Mehren M., Blanke C.D., Van den Abbeele A.D., Eisenberg B., Roberts P.J., Heinrich M.C., Tuveson D.A., Singer S., Janicek M. (2002). Efficacy and safety of imatinib mesylate in advanced gastrointestinal stromal tumors. N. Engl. J. Med..

[248] Blanke C.D., Demetri G.D., von Mehren M., Heinrich M.C., Eisenberg B., Fletcher J.A., Corless C.L., Fletcher C.D., Roberts P.J., Heinz D. (2008). Long-term results from a randomized phase ii trial of standard- versus higher-dose imatinib mesylate for patients with unresectable or metastatic gastrointestinal stromal tumors expressing kit. J. Clin. Oncol..

[249] Antonescu C.R., Besmer P., Guo T., Arkun K., Hom G., Koryotowski B., Leversha M.A., Jeffrey P.D., Desantis D., Singer S. (2005). Acquired resistance to imatinib in gastrointestinal stromal tumor occurs through secondary gene mutation. Clin. Cancer Res..

[250] Demetri G.D., Garrett C.R., Schoffski P., Shah M.H., Verweij J., Leyvraz S., Hurwitz H.I., Pousa A.L., Le Cesne A., Goldstein D. (2012). Complete longitudinal analyses of the randomized, placebo-controlled, phase iii trial of sunitinib in patients with gastrointestinal stromal tumor following imatinib failure. Clin. Cancer Res..

[251] Demetri G.D., Reichardt P., Kang Y.K., Blay J.Y., Rutkowski P., Gelderblom H., Hohenberger P., Leahy M., von Mehren M., Joensuu H. (2013). Efficacy and safety of regorafenib for advanced gastrointestinal stromal tumours after failure of imatinib and sunitinib (grid): An international, multicentre, randomised, placebo-controlled, phase 3 trial. Lancet.

[252] Tamborini E., Pricl S., Negri T., Lagonigro M.S., Miselli F., Greco A., Gronchi A., Casali P.G., Ferrone M., Fermeglia M. (2006). Functional analyses and molecular modeling of two c-kit mutations responsible for imatinib secondary resistance in gist patients. Oncogene.

[253] Chen L.L., Holden J.A., Choi H., Zhu J., Wu E.F., Jones K.A., Ward J.H., Andtbacka R.H., Randall R.L., Scaife C.L. (2008). Evolution from heterozygous to homozygous kit mutation in gastrointestinal stromal tumor correlates with the mechanism of mitotic nondisjunction and significant tumor progression. Mod. Pathol..

[254] Agaram N.P., Wong G.C., Guo T., Maki R.G., Singer S., Dematteo R.P., Besmer P., Antonescu C.R. (2008). Novel v600e braf mutations in imatinib-naive and imatinib-resistant gastrointestinal stromal tumors. Genes Chromosom. Cancer.

[255] Mahadevan D., Cooke L., Riley C., Swart R., Simons B., Della Croce K., Wisner L., Iorio M., Shakalya K., Garewal H. (2007). A novel tyrosine kinase switch is a mechanism of imatinib resistance in gastrointestinal stromal tumors. Oncogene.

[256] Sakurama K., Noma K., Takaoka M., Tomono Y., Watanabe N., Hatakeyama S., Ohmori O., Hirota S., Motoki T., Shirakawa Y. (2009). Inhibition of focal adhesion kinase as a potential therapeutic strategy for imatinib-resistant gastrointestinal stromal tumor. Mol. Cancer Ther..

[257] Tarn C., Rink L., Merkel E., Flieder D., Pathak H., Koumbi D., Testa J.R., Eisenberg B., von Mehren M., Godwin A.K. (2008). Insulin-like growth factor 1 receptor is a potential therapeutic target for gastrointestinal stromal tumors. Proc. Natl. Acad. Sci. USA.

[258] Li F., Huynh H., Li X., Ruddy D.A., Wang Y., Ong R., Chow P., Qiu S., Tam A., Rakiec D.P. (2015). Fgfr-mediated reactivation of mapk signaling attenuates antitumor effects of imatinib in gastrointestinal stromal tumors. Cancer Discov..

